# Non-polio enterovirus infection and electrophysiological changes in human iPSC-derived neural networks

**DOI:** 10.1016/j.ebiom.2026.106201

**Published:** 2026-03-12

**Authors:** Feline F.W. Benavides, Syriam Sooksawasdi Na Ayudhya, Ashley K. Pereirinha da Silva, Mark A. Power, Willemijn F. Rijnink, Auriane Deguergue, Bjoern Meyer, Femke M.S. de Vrij, Debby van Riel, Kristina Lanko, Lisa Bauer

**Affiliations:** a Department of Viroscience, Erasmus University Medical Center, Rotterdam, the Netherlands; b Medical Faculty, Institute of Medical Microbiology and Hospital Hygiene, Otto-von-Guericke-University Magdeburg, Magdeburg, Germany; c Department of Psychiatry, Erasmus University Medical Center, Rotterdam, the Netherlands; d Department of Clinical Genetics, Erasmus University Medical Center, Rotterdam, the Netherlands

**Keywords:** Non-polio enteroviruses, Neurovirulence, Neurotropism, Micro-electrode array, Neural networks

## Abstract

**Background:**

The non-polio enteroviruses (NPEV) enterovirus D68 (EV-D68) and enterovirus A71 (EV-A71) are highly prevalent and considered pathogens of increasing health concern due to their neurotropic potential. Severe neurological complications of usually mild and self-limiting NPEV infections include meningitis, encephalitis, and acute flaccid paralysis, especially in children and immunocompromised patients. Despite clinical burden, the underlying neuropathogenesis of EV-D68 and EV-A71 remains poorly understood. In particular, the impact of the infection on neural function has not been clearly elucidated.

**Methods:**

We investigate the replication kinetics, cellular tropism, pro-inflammatory cytokine responses, and electrophysiological effects of EV-D68 and EV-A71 infection in a physiologically relevant human pluripotent stem cell-derived neural co-culture model, consisting of excitatory neurons and astrocytes using a micro-electrode array platform.

**Findings:**

All NPEV replicated efficiently in the neural co-cultures and infection was detected in both neurons and astrocytes. Both EV-D68 and EV-A71 infection resulted in decreased neural activity in the co-cultures, with the EV-D68 clade A2/2018 inducing the most rapid and robust negative effect on neural co-cultures, followed by EV-D68 clade B3/2019. Despite the lack of release of infectious virus particles of EV-D68 B3/2019 in the supernatant, the infection could spread in the cultures and reduce neurotransmission. Higher viral load of EV-A71 did not result in enhanced impairment of neural function.

**Interpretation:**

Our results demonstrate that neurotropic NPEVs lead to disruption of spontaneous neural activity in a virus-specific manner, which does not correlate with their replication efficiency.

**Funding:**

The Netherlands Organisation for Health Research, Development and the 10.13039/501100003246Dutch Research Council, the Netherlands Organ-on-Chip Initiative.

**Research in context**

**Evidence before this study**

(Re)-emerging non-polio enteroviruses (NPEV) are widespread and increasingly recognised as important pathogens of public health concern. We searched PubMed using the terms (“non-polio enteroviruses” OR “enteroviruses”) AND (“encephalitis” OR “neuron” for articles without language restriction published between Jan 1, 2000, and Jan 31, 2025 and identified 488 publications. In most cases, NPEV infections manifest as mild, self-limiting illness, such as fever or respiratory symptoms. However, a subset of infections can progress to severe and sometimes life-threatening complications, including meningitis, encephalitis, and acute flaccid paralysis, especially in children and immunocompromised patients. Despite the significant global burden of neurological disease linked to enterovirus D68 (EV-D68) and enterovirus A71 (EV-A71), the molecular and cellular mechanisms underlying the neuropathogenesis remains poorly understood and only one publication investigated the neural network function in an *in vitro* infection model.

**Added value of this study**

This study investigates NPEV replication in a physiologically relevant human induced pluripotent stem cell (hPSC)-derived neural network model, consisting of neurons and astrocytes. The NPEVs strains used in this study infected both neurons and astrocytes. Both EV-D68 strains, and EV-A71 resulted in an overall reduction of spontaneous activity of the neural co-cultures, measured by a multi-electrode array platform. The EV-D68 clade A2/2018 induced the most rapid and robust negative effect on the neurotransmission of the neural co-cultures, followed by EV-D68 clade B3/2019. Despite the lack of viral release of infectious virus particles of EV-D68 B3/2019 in the supernatant, the infection could spread in the cultures and reduced neurotransmission. Higher viral load and broader tropism of EV-A71 did not result in enhanced impairment of neural function. We evaluated the pro-inflammatory cytokine response and detected the secretion of interleukin-6 and -8 in infected cultures. Our results demonstrate that neurotropic NPEVs lead to disruption of spontaneous neural activity in a virus-specific manner, which does not correlate with their replication efficiency.

**Implications of all the available evidence**

This study provides insights into the neuropathogenesis of NPEVs, demonstrating that EV infections have direct profound effects on the neural network activity. Using a physiologically relevant hPSC-derived neural *in vitro* model provides a link between NPEV replication and disturbances in the brain homoeostasis and functionality. While the clinical pictures of NPEV induced neurological sequelae share similarities, on the cellular level different NPEVs employ distinct neuropathogenic strategies. Potential future treatment strategies might involve prevention of neuroinvasion by vaccination or timely antiviral administration, while short-term management could focus on therapies supporting and improving neural function.

## Introduction

The genus *Enterovirus* within the family *Picornaviridae* comprises over 250 clinically relevant virus serotypes that circulate worldwide.^1^, ^2^, ^3^, ^4^ Enterovirus infections are often unnoticed and self-resolving; however, they can cause serious illnesses that are associated with major, sometimes life-threatening complications, especially in infants, young children, and immunocompromised individuals.^5^, ^6^, ^7^, ^8^ Enterovirus infections display a diverse clinical spectrum, including upper and lower respiratory disease, gastroenteritis, hand-foot-and-mouth disease, conjunctivitis, myocarditis, severe neonatal sepsis-like diseases, aseptic meningitis, encephalitis, acute flaccid paralysis (AFP), and acute flaccid myelitis (AFM).^9^^,^^10^ The incidence of encephalitis in non-polio enterovirus (NPEV) infection is reported to be about 3% and is associated with high morbidity. Furthermore, in retrospective or epidemiological studies it is suggested that 10–58% of diagnosed encephalitis/meningitis cases have an underlying enterovirus infection.^11^, ^12^, ^13^ In recent years, NPEVs, in particular enterovirus D68 (EV-D68) and EV-A71, have been considered pathogens of increasing health concern since their (re)-emergence is associated with the development of neurological complications.^14^, ^15^, ^16^ EV-D68 and EV-A71 are classified into several major genotypes and globally multiple EV-A71 genotypes are circulating,^17^ while among EV-D68 viruses, the subclades A2 and B3 are currently most prevalent.^18^, ^19^, ^20^ The spectrum of neurological complications for these two pathogens largely coincides and includes aseptic meningitis, encephalitis, and AFM with some differences in clinical representations.^8^^,^^10^^,^^21^

After replication in the primary infection sites—the gastrointestinal tract for EV-A71 and the respiratory tract for EV-D68—the viruses can get access to the CNS through multiple but not mutually exclusive pathways (e.g. haematogenous spread or infection of peripheral/cranial neurons). *In vitro* studies suggest that EV-D68 and EV-A71 disseminate to the CNS via the bloodstream by disrupting the blood–brain barrier through for example direct infection of brain endothelial cells.^22^ Another option is the so-called Trojan horse mechanism, where viruses exploit blood cells to cross the blood–brain barrier. It has been shown that EV-A71 uses this mechanism; it is unclear whether EV-D68 can invade the CNS in the same way, but the infection of blood-derived immune cells has been demonstrated *in vitro*.^23^^,^^24^ In the brain, MRI studies revealed hyperintensity lesions induced by EV-D68 and EV-A71 infection, and immune responses have been detected in different brain regions, as well as the spinal cord in patients with CNS involvement.^25^^,^^26^ EV-A71 antigen was detected in neuronal cell bodies and astrocytes across different regions,^26^, ^27^, ^28^ but for EV-D68, limited pathological data are available. In the spinal cord, viral antigen and RNA of EV-D68, and EV-A71 antigen have been detected in motor neurons of the anterior horn.^27^^,^^29^^,^^30^ Among other symptoms, patients with enterovirus-induced CNS complications present with lethargy, cognitive impairment, or seizures, indicating disruption of normal neural homoeostasis, and function.^31^^,^^32^

Neurotransmission plays a critical role in cognitive functions, and disruptions can lead to deficits, including impairments in learning, memory, and executive function. It has been shown that viral infection can result in changes in neurotransmission, leading to changes in behaviour.^33^, ^34^, ^35^, ^36^ Electrophysiology is a widely used tool to investigate neurotransmission and can be used both *in vitro* and *in vivo*. A micro-electrode array (MEA) platform is a useful and easy tool to investigate the functional consequences of viral infections on neurotransmission in a continuous manner. Recently, it has been shown that EV-D68 infection of primary rat cortical neurons resulted in reduced neurotransmission measured by MEA,^37^ but research in human-derived models is lacking. Whether EV-A71 also impairs neurotransmission is currently not understood. Induced excitatory neurons generated through Neurogenin-2 (Ngn2) overexpression provide a fast and reliable model system for studying neural function.^38^, ^39^, ^40^ These Ngn2-induced neurons, when co-cultured with astrocytes, are capable of forming functional synapses and display robust electrophysiological properties. This enables us to study spontaneous neuronal activity and assess functional network connectivity in a physiologically relevant human model.^38^^,^^40^, ^41^, ^42^

In this study, we investigated the functional consequences of infection with clinically prevalent enteroviruses in a human pluripotent stem cell (hPSC)-derived neural co-culture model. To this end, we employed networks of neural co-cultures to characterise neurotropism, replication kinetics and neural activity upon enterovirus-D68 and enterovirus-A71 infection on a MEA platform to better understand the underlying mechanisms of neurological complications associated with non-polio enterovirus infections.

## Methods

### Cells and reagents

Hela-R19 cells (RRID:CVCL_M763, kindly provided by Johan Neyts from the Rega Institute at the KU Leuven) and HEK293T cells (RRID:CVCL_0063 obtained from ATCC Cat# CRL-3216) were maintained in Dulbecco's Modified Eagle's medium (DMEM; Capricorn Scientific, Frankfurt am Main, Germany) supplemented with 10% dialysed foetal bovine serum (FBS, Sigma–Aldrich, St. Louis, MO, USA), 2 mM l-glutamine (Capricorn Scientific) and 1% penicillin/streptomycin (Capricorn Scientific) at 37 °C with 5% CO_2_. Hela-R19 cells were used for generating virus stocks as well as for viral titration experiments. HEK293T cells were used for transfection of infectious clone plasmids. Medium was refreshed every 2–4 days, and cells were passaged at >80% confluence using PBS and trypsin–EDTA (Capricorn Scientific). Cells were regularly checked for presence of mycoplasma. The compounds (*S*)-fluoxetine (SFX, Sigma Aldrich), forskolin (Tocris) and rolipram (Tocris) were dissolved in DMSO at 10 mM, 25 mM and 50 mM stock concentration, respectively. Tetrodotoxin (TTX, Tocris) was dissolved in citric acid buffer pH 4.8 at a stock concentration 3 mM. Polyinosinic: polycytidylic acid (polyI:C) was purchased from InvivoGen and dissolved in water to a stock concentration of 1 mg/mL.

### Viruses

EV-D68 strains included in this study were provided by Adam Meijer at the National Institute of Public Health and the Environment (RIVM), Bilthoven, The Netherlands. EV-D68 viruses were propagated on RD cells at 33 °C in 5% CO2. EV-D68 viruses from different clades were included in this study, virus reference number and accession number are as follow: clade A/2012 (4311200821; Accession Number: MN954536), A2/2018 (4311400720; Accession Number: MN954537), B1/2013 (4311300117; Accession number MN954538), B2/039 (4311201039; Accession Number: MN954539), B3/2019 (3101900710; Accession Number: MN726799) and Fermon (Accession Number: AY426531). All virus stocks were Sanger sequenced. Recombinant viruses from EV-A71 Sep006 (Accession Number: MG208882) and EV-D68 Fermon were obtained by transfecting the plasmids of the infectious clones pCAGGS-EVA71-Sep006 and pCAGGS_EV-D68-Fermon in HEK293T cells. After obtaining full cytopathic effect in HEK293T cells, the virus was one more time passaged in Hela-R19 cells.

### Human induced pluripotent stem cells

Commercially available human pluripotent stem cells (hPSC) WTC-11 (Coriell no. GM25256, RRID:CVCL_Y803, obtained from the Gladstone Institute, San Francisco, CA, USA, were used to generate astrocytes. WTC-11 hPSCs were maintained in hPSCs medium (Table 1), released with Accutase (Life Technologies), and grown in Matrigel (Corning)-coated 6-well plates. Medium was refreshed every other day, and cells were cultured at 37 °C and 5% CO2.

**Table 1:** Overview of media used for differentiation and maintaining of stem cells.

Name	Reagents with final concentration	Manufacturer
hPSC medium	Stemflex medium	ThermoFisher Scientific
	100 IU/mL penicillin	Lonza
	100 μg/mL streptomycin	Lonza
	10 μl/mL fresh RevitaCell	ThermoFisher Scientific
Differentiation medium	Advanced DMEM/F12 medium	ThermoFisher Scientific
	100 IU/mL penicillin	Lonza
	100 μg/mL streptomycin	Lonza
	0.1 mM non-essential amino acids	Lonza
	1% N2 supplement	ThermoFisher Scientific
	10 ng/mL fresh Human Recombinant Neurotrophin-3 (NT3)	Stemcell Technologies
	10 ng/mL fresh brain-derived neurotrophic factor (BDNF)	Prospecbio
	200 ng/mL fresh laminin	Corning
	4 μg/mL fresh Doxycycline	Sigma
Ngn2 medium	Neurobasal medium	ThermoFisher Scientific
	100 IU/mL penicillin	Lonza
	100 μg/mL streptomycin	Lonza
	2 mM glutamine	Lonza
	2% B27 minus RA supplement	ThermoFisher Scientific
	10 ng/mL fresh Human Recombinant Neurotrophin-3 (NT3)	Stemcell Technologies
	10 ng/mL fresh brain-derived neurotrophic factor (BDNF)	Prospecbio
	4 μg/mL fresh Doxycycline (DOX)	Sigma
Neural progenitor cells medium	Advanced DMEM/F12 medium	ThermoFisher Scientific
	100 IU/mL penicillin	Lonza
	100 μg/mL streptomycin	Lonza
	1% N2 supplement	ThermoFisher Scientific
	2% B27 minus RA supplement	ThermoFisher Scientific
	1 μg/mL laminin	Corning
	20 ng/mL fresh Fibroblast Growth Factor (FGF)	Merck
Astrocytes medium	Advanced DMEM/F12 medium	ThermoFisher Scientific
	100 IU/mL penicillin	Lonza
	100 μg/mL streptomycin	Lonza
	1% N2 supplement	ThermoFisher Scientific
	2% B27 minus RA supplement	ThermoFisher Scientific
	1 μg/mL laminin	Corning
	10 ng/mL fresh Bone Morphogenetic Protein 4 (BMP4)	Prospec
	10 ng/mL fresh Leukemia Inhibitory Factor (LIF)	Tebu-bio

### Ethics

Ethical approval for the use of the WTC-11 hiPSC line was obtained from the San Francisco General Hospital Panel. Voluntary Informed consent for the use of WTC-11 for research purpose was given under the study number 10-02521 by the GESCR committee, University of California and details can be found under the following link: https://hpscreg.eu/cell-line/UCSFi001-A.

### Differentiation of hiPSC into NGN2 neuron-astrocyte co-cultures

WTC-11 hPSCs were directly differentiated into excitatory cortical layer 2/3 neurons by overexpression of Ngn2 using an adapted protocol for inducible overexpression in the presence of doxycycline, as previously described.^38^^,^^39^^,^^43^ In short, coverslips or MEA plates were coated with poly-l-ornithine (Sigma, 100 μg/mL) for 1 hr at RT in the dark. Then coverslips or MEA plates were washed three times with water and dried. Coverslips or MEA plates were then coated with Matrigel (Corning, 10 μl/mL resuspended in KO DMEM) for 1 h at 37 °C. After incubation, the hPSCs were plated in hPSC medium (Table 1) supplemented with 4 μg/mL fresh doxycycline (Sigma). The next day, the medium was refreshed with differentiation medium (Table 1). In order to guarantee the formation of functional synapses and thus functional synaptic plasticity within the network, hPSC-derived astrocytes were added on day 3. hPSC-derived astrocytes were differentiated from hPSCs through neural progenitor cells (NPCs) as previously described.^40^ hPSC-derived astrocytes were added to the culture in a 1:1 ratio. The medium was refreshed the day after with Ngn2 medium (Table 1). Every other day, half of the medium was refreshed until day 21. During maintenance, and differentiation of hPSC-derived astrocytes and NPC, whole medium was refreshed with astrocytes or NPC medium (Table 1) every other day and cells were split once per week. All cells were kept at 37 °C and 5% CO_2_.

### Virus infection

Virus infections for the replication kinetic were performed by incubating hPSC-derived neural co-cultures with virus at a multiplicity of infection (MOI) of 0.1 at 37 °C in 5% CO_2_ for 1 h for viral titration or immunofluorescent staining. Subsequently, the inoculum was removed, the cultures were washed once with PBS after which half fresh and half old medium was added to the hPSC-derived neural co-cultures. Supernatants for virus titration were collected at the indicated time points. For determining cytotoxicity, apoptosis and for MEA measurements, hPSC-derived neural co-cultures were incubated with virus at MOI 1, inoculum was removed after 1 h, washed once with PBS and old medium was added 1:1 to the neural co-cultures. Medium was refreshed half every other day.

### Virus titration

Virus titres were determined by endpoint dilution on a subconfluent layer of Hela-R19 cells. Briefly, 10-fold serial dilutions of samples were titrated on Hela-R19 cells and incubated at 33 °C in 5% CO_2_. At day 5, virus titres were determined by visual inspection of cytopathic effect. Viral titres were calculated according to the method of Reed and Munch and expressed as 50% tissue culture infective dose (TCID50).^44^

### Multiplexed bead-assay for cytokine profiling

Cytokines were measured using the LEGENDplex™ Human Anti-Virus Response Panel (BioLegend). The kit was used according to manufacturer's manual and cytokines were measure on a flow cytometer (BD FACSLyric) and analysed with the software LEGENDplex™ Data Analysis Software Suite Version 2025-05-01 from Qognit.

### Immunofluorescence staining

At indicated time points, hPSC-derived neural co-cultures were fixed with 10% formalin for 15 min and permeabilized with 1% triton (Sigma; T8787) in PBS for 15 min. Cells were blocked with 5% bovine serum albumin (BSA; Aurion) for 30 min after which cells were incubated with primary antibodies for 1 h. Cells were washed twice with washing buffer (PBS with 0.1% BSA) and incubated with secondary antibodies for 1 h at room temperature. Primary antibodies and secondary antibodies with used concentrations can be found in Table 2. The cells were washed three times with washing buffer and afterwards nuclei were stained using the dye Hoechst (1:1000, Invitrogen, H3570) for 10 min at room temperature. Cells were washed once with distilled water and mounted on glas slides. Samples were processed using a Zeiss LSM 700 laser scanning microscope. All images were processed using Zen 2010 software and Image J.

**Table 2:** Concentration of used antibodies.

Antibody	Antibody	Concentration	Manufacturer	Catalogue no.
Primary	Anti-dsRNA Antibody, clone rJ2, RRID:AB_2922431	1:400 dilution	Jena Bioscience	RNT-SCI-10010200
	Rabbit anti-EV-D68 VP1, RRID:AB_2886609	10 μg/mL	GeneTex	132313
	Guinea pig anti-MAP2 RRID:AB_2138181	2% v/v	Synaptic Systems	188004
	Mouse anti-GFAP, RRID:AB_396365	2.5 μg/mL	BD Pharma	556327
	Chicken anti-GFAP, RRID:AB_304558	10 μg/mL	Abcam	AB-4674
	Mouse α-Homer1 RRID:AB_2619855	13.33 μg/mL	Synaptic systems	160011
	Rabbit α-Synapsin I, RRID:AB_11042000	10 μg/mL	Synaptic systems	106103
	Rabbit Cleaved Caspase-3, RRID:AB_2341188	0.5 μg/mL	Cell Signalling	9661
Secondary	Donkey anti-rabbit AF488, RRID:AB_2535792	20 μg/mL	Invitrogen	A21206
	Donkey anti-mouse AF555, RRID:AB_2536180	10 μg/mL	Invitrogen	A31570
	Donkey anti-guinea pig AF647 RRID:AB_2340476	2.5 μg/mL	Jackson Immuno research	706-605-148
	Donkey anti-chicken AF555, RRID:AB_2921071	2 μg/mL	Invitrogen	A78949

### LDH-assay

Cell death was quantified by measuring lactate dehydrogenase (LDH) released into the culture supernatant using the CytoTox 96® Non-Radioactive Cytotoxicity Assay (G1780; Promega) according to the manufacturer's protocol. Briefly, culture supernatant was incubated for 30 min with the substrate mix and after adding the stop solution the optical density (490 nm) was recorded with a Varioscan (ThermoFisher). A maximal LDH-release control was established by lysing uninfected neural co-cultures with 10 μl of 10× Lysis Solution per 100 μl of medium for 30 min. The mean OD_490_ value from lysed samples was defined as 100% LDH. All experimental LDH values were then expressed as a percentage of this maximum. To represent the level of viable, non-lysed cells, the LDH values from mock-treated cultures were subsequently normalised and set to 100% live cells.

### Image quantification

To quantify infection percentage, 10 images were taken from coverslips from three independent experiments. The total staining of Hoechst, glial fibrillary acidic protein (GFAP) and microtubule-associated protein 2 (MAP2) was annotated per image using the pixel classifier function in QuPath 0.6.0^45^ with a Gaussian filter. The threshold values to determine positive surface area can be found in Table 3. The total positive area was then annotated and from this annotation the pixel classifier function was run again to determine the positive area of virus staining (dsRNA). In addition, staining of GFAP or MAP2 was annotated in a similar fashion to determine the positive area of virus staining per cell type. Individual data points were plotted with mean ± SEM.

**Table 3:** Chosen threshold values used in QuPath to determine positive areas.

Experiment	Timepoint	Staining (fluorophore)	Threshold
Infection	24 and 72 hpi	Hoechst	40
	24 and 72 hpi	dsRNA (Alexa488)	20
	24 and 72 hpi	GFAP (Alexa555)	20
	24 and 72 hpi	MAP2 (Alexa647)	10
Cleaved caspase-3 (MAP2 images)	24 hpi	Hoechst	40
	24 hpi	dsRNA (Alexa488)	20
	24 hpi	Cleaved caspase-3 (Alexa555)	30
	24 hpi	MAP2 (Alexa647)	5
Cleaved caspase-3 (GFAP images)	24 hpi	Hoechst	40
	24 hpi	dsRNA (Alexa488)	20
	24 hpi	GFAP (Alexa555)	35
	24 hpi	Cleaved caspase-3 (Alexa647)	10
Cleaved caspase-3 (MAP2 images)	72 hpi	Hoechst	40
	72 hpi	dsRNA (Alexa488)	20
	72 hpi	Cleaved caspase-3 (Alexa555)	40
	72 hpi	MAP2 (Alexa647)	5
Cleaved caspase-3 (GFAP images)	72 hpi	Hoechst	40
	72 hpi	dsRNA (Alexa488)	20
	72 hpi	GFAP (Alexa555)	35
	72 hpi	Cleaved caspase-3 (Alexa647)	40

To quantify the expression of cleaved caspase-3, 10 images were taken from coverslips from three independent experiments. The total staining area of Hoechst and MAP2 or Hoechst and GFAP was determined by annotating the positive area that was measured with the pixel classifier function in QuPath 0.6.0 (threshold values in Table 3). The positive area of cleaved caspase-3 was then determined using the pixel classifier function in QuPath 0.6.0 (threshold values in Table 3) on the annotated area of Hoechst and GFAP or Hoechst and MAP2. Individual data points were plotted with mean ± SEM.

### MEA recordings of neural activity in Ngn2 neurons co-cultured with astrocytes

Neural co-cultures were plated on 24-well CytoView MEA plates (Axion Biosystems). Plates were acclimatized for at least 5 min before neural activity and viability were measured for 5 min using the Axion Biosystems Maestro MEA at 37 °C and 5% CO_2_. Data analysis was performed using AxIs software (Axion Biosystems Inc.). Covered electrodes were defined as electrodes with a minimum resistance of 18 kΩ. We excluded wells for further analysis if a well reached ≤5 covered electrodes at any point during the experiment. Active electrodes were defined as electrodes with a minimum of five spikes per minute. We excluded wells for analysis if a well had ≤5 active electrodes at the baseline recording for further analysis. The threshold for spike detection was defined as ≥6-fold the standard deviation (SD) of the root mean square noise. The threshold for burst detection was defined as >5 spikes within a time window of 100 ms. The threshold for network burst detection was defined as >50 spikes within a time window of 100 ms, with a minimum of 50% participating electrodes per well.

For stimulation and inhibition experiments, pre-warmed drugs were infused in the medium in an amount of maximum 10% of the medium. Before adding drugs, a baseline recording was performed. For stimulation, protocol was followed as described before.^46^ In short, 50 μM forskolin and 0.1 μM rolipram were diluted in Ngn2 medium. The medium containing forskolin and rolipram was added to the cells and were incubated for half an hour, before washing it out. For inhibition, a protocol was followed as described before.^47^ 10 μM SFX was diluted in Ngn2 medium and added to the neural co-cultures. Additionally, to completely block neurotransmission, neural co-cultures were treated with the sodium-channel blocker TTX with a final concentration of 0.5 μM.^39^ As negative control, medium containing the same amount of DMSO was included.

### PCA analysis and heatmap generation

MEA datasets from neural co-cultures (mock-inoculated or inoculated with EV-D68 A2/2018, EV-D68 B3/2019, or EV-A71 Sep006) were first normalised to baseline recordings obtained prior to inoculation. Only data that met the inclusion criteria described earlier for MEA recordings were used. Any experiment that contained *Not Available* values throughout any of the chosen MEA variables (Table 4) was excluded for principle component analysis (PCA), to ensure biological relevance and integrity of the dataset. Data were mean-centred and scaled to unit variance (z-score normalisation) for each variable (feature) across the analysed samples. Z-scoring was used to place features measured on different scales onto a comparable range for variance- and distance-based multivariate analyses (PCA and hierarchical clustering), as done in prior MEA studies that applied standard score normalisation before PCA.^49^^,^^50^ PCA was performed using the prcomp function in R (v4.5.1; RStudio) with the stats package, and results were visualised with ggplot2 (v3.5.2). The first two principal components (PC1 and PC2) were plotted with a 95% prediction ellipse. Heatmaps were generated using pheatmap (v1.0.12) on the z-scored data matrix. Hierarchical clustering was performed on both samples and features using Euclidean distance and complete linkage, with dendrogram-based ordering. Data processing used readxl (v1.4.3) and dplyr (v1.1.4). Colour intensity reflects a positive or negative effect on neural activity, with white to red indicating a more positive effect.

**Table 4:** Output MEA variables that were chosen for Principal Component Analysis as defined by the Axion Neural Metric guide.

MEA variables	Definition
Mean firing rate	Total number of spikes divided by the duration of the recording
Mean inter-spike interval (ISI) coefficient of variation	Measure of spike regularity: the coefficient of variation (standard deviation/mean) of the inter-spike interval
Number of active electrodes	Number of electrodes with activity greater than 5 spikes per minute (inclusion criteria)
Number of bursting electrodes	Number of electrodes within the well with bursts that are included in analysis (inclusion criteria for bursts: >5 spikes within 100 ms/electrode)
Burst duration	Time from the first spike to last spike in a single-electrode burst
Number of spikes per burst	Number of spikes in a single-electrode burst
Mean ISI within burst	ISI time between spikes, for spikes in a single-electrode burst
Median ISI within burst	Median ISI time between spikes, for spikes in a single-electrode burst
Inter-Burst Interval (IBI)	Time elapsed between the end of one burst and the beginning of the next
Burst frequency	Total number of single-electrode bursts divided by the duration of the recording
IBI coefficient of variation	Measure of burst regularity: the coefficient of variation (standard deviation/mean) of the inter-burst interval
Burst percentage	The number of spikes in single-electrode bursts divided by the total number of spikes, multiplied by 100
Network burst frequency	Total number of network bursts divided by the duration of the recording (included when ≥50% electrodes are participating in the burst and the burst have >50 spikes within 100 ms)
Network burst duration	Average time from the first spike to last spike in a network burst
Number of spikes per network burst	Number of spikes in a network burst
Number of electrodes participating in burst	Number of electrodes with activity during a network burst
Number of spikes per network burst per channel	Number of spikes in a network burst divided by the number of electrodes participating in that burst
Network burst percentage	The number of spikes in network bursts divided by the total number of spikes, multiplied by 100.
Network ISI coefficient of variation	Measure of spike regularity across network activity: the coefficient of variation (standard deviation/mean) for the inter-spike interval for all spikes on all electrodes in a well
Network IBI coefficient of variation	Measure of network burst regularity: the coefficient of variation (standard deviation/mean) for the inter-network burst interval, the time between network bursts.
Network normalised duration IQR	Measure of network burst duration regularity: interquartile range of network burst durations.
Area under normalised cross-correlation	Area under the well-wide pooled inter-electrode cross-correlation normalised to the auto-correlations. Higher areas indicate greater synchrony
Width at half height of normalised cross-correlation	Measure of network synchrony: distance along the x-axis (phase lag) from left half height to right half height (probability) of the normalised cross-correlogram.
Synchrony index	A unitless measure of synchrony between 0 and 1.^48^ Values closer to 1 indicate higher synchrony.
Resistance–Avg (kOhms)	Resistance is a measure of viable cell coverage over the electrode. Higher values indicate more intact cells are attached to the electrode (inclusion when resistance >18 kΩ)
Number of covered electrodes	Covered electrodes are defined as electrodes with resistance greater than 18 kΩ

### Statistical analysis and figures

For MEA experiments at least four independent experiments (derived from four independent differentiations) performed in at least 6 biological replicates were performed. Data obtained from viral growth kinetics, immunofluorescence staining and cytokines assay are derived from three independent experiments (each experiment an independent differentiation) performed in biological triplicates. A Kolmogorov–Smirnov test showed that the data are normally distributed. Statistical differences between experimental groups were determined as described in the figure legends. *P* values of  ≤0.05 were considered significant. Graphs and statistical tests were made with GraphPad Prism version 10.5.0 and R-Studio Version 2023.12.0 + 369. Figures were prepared with ImageJ 1.54f, and Adobe Illustrator 27.8.1.

### Role of the funding source

The funders of the study had no role in the study design, data collection, data analysis, data interpretation, or writing of the report.

## Results

### Characterisation of hPSC-derived neuron and astrocyte co-cultures to study the neurovirulence of NPEVs

**Fig. 1:**
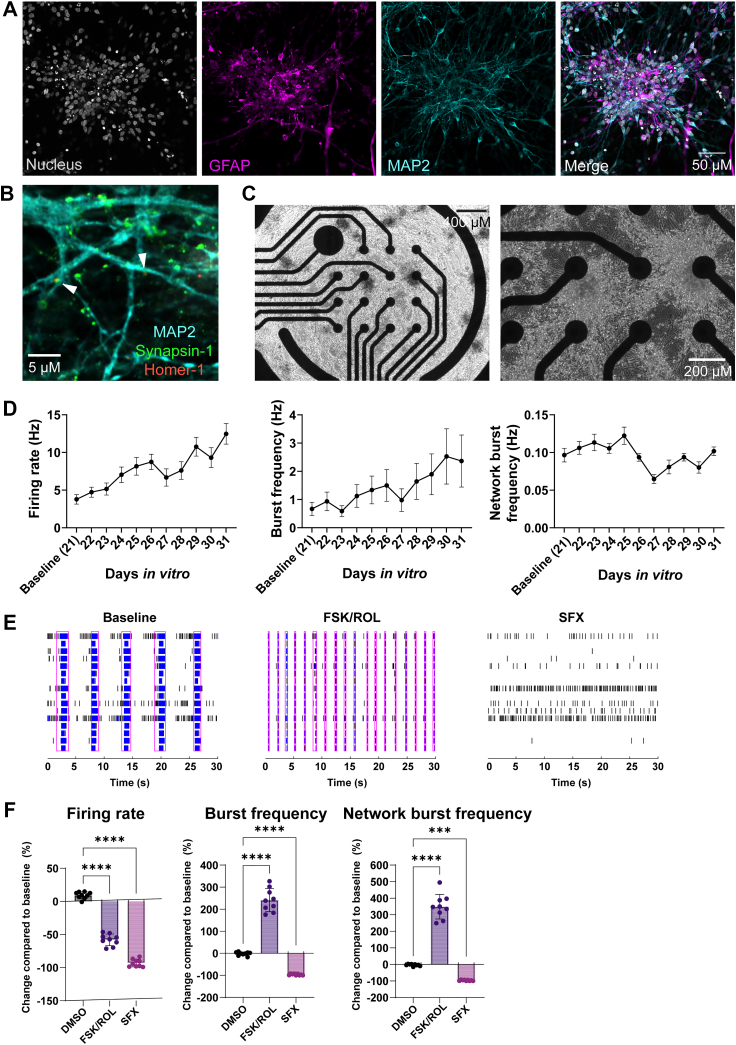
**Ngn2-expressing neurons co-cultured with astrocytes form functional neural networks and respond to stimulation and inhibition.** hPSC-derived Ngn2-expressing neurons were co-cultured with hPSC-derived astrocytes and matured for 21 DIV. (A) Immunofluorescent staining was performed with astrocytic-specific marker GFAP (magenta) and the neuron-specific marker MAP2 (cyan). Cells were counterstained with the nuclear dye Hoechst at DIV 21. (B) Immunofluorescent staining for functional synapses was performed against MAP2 (cyan), synapsin 1 (green, marker for pre-synaptic density), and Homer 1 (red, marker for post-synaptic density) at DIV 22. (C) Brightfield images of neural co-cultures plated on micro-electrode array plates to measure neural activity at day 21. Data shown are representative pictures of at least 2 independent experiments (Panel A–C). (D) Parameters for neural activity of Ngn2 neural co-cultures between DIV 21 and 31: firing rate, burst frequency, and network burst frequency. Data are depicted as mean ± SEM and are derived from eight independent experiments (*n* = 6 per experiment, unless datapoints were excluded based on exclusion criteria, see Material and Methods). (E) Raster plots of neural co-cultures were stimulated with 50 μM FSK and 0.1 μM ROL or inhibited with 10 μM SFX. (F) Firing rate, burst frequency, and network burst frequency measured after stimulation or inhibition are shown. Data are depicted as mean ± SD. Data are derived from two independent experiments (*n* = 9 per group, unless datapoints were excluded based on exclusion criteria, see Material and Methods). Statistical significance was determined with a One-Way ANOVA, comparing the DMSO treatment to FSK/Rol and SFX treatment. Asterisks indicate statistical significance (∗*P* < 0.05, ∗∗*P* < 0.01, ∗∗∗*P* < 0.001, ∗∗∗∗*P* < 0.0001). Abbreviations: hPSC, human induced pluripotent stem cells; Ngn2, Neurogenin-2; DIV, days *in vitro*; GFAP, glial fibrillary acidic protein; MAP2, microtubule-associated protein 2; SEM, standard error of the mean; DMSO, dimethylsulfoxide; FSK, forskolin; ROL, rolipram; SFX, (*S*)-fluoxetine; SD, standard deviation.

To study the effect of enterovirus infection on neural networks, we employed a rapid differentiation protocol that yields hPSC-derived excitatory glutamatergic cortical neurons by overexpressing the transcription factor Ngn2 as previously described.^38^^,^^39^ We supplemented the Ngn2-induced neurons with hPSC-derived astrocytes to ensure neuron survival and maturation (Supplementary Fig. S1A). Immunofluorescence staining revealed that the cultures contained GFAP^+^ astrocytes and MAP2^+^ neurons at 21 *days in vitro* (DIV) (Fig. 1A). Furthermore, immunofluorescence staining of pre- and postsynaptic density proteins showed that these cultures establish synaptic connections (Fig. 1B, Supplementary Fig. S1B). We next assessed neural network activity of the Ngn2 neural co-cultures using a MEA system (Fig. 1C, Supplementary Fig. S1C). The neural co-cultures exhibit spontaneous neural activity, displayed by firing rate, burst frequency, and network burst frequency between DIV 21 and 31 (Fig. 1D). To probe the activity of the cultures, we stimulated the neural co-cultures with 50 μM forskolin (FSK) and 0.1 μM rolipram (ROL) to induce long-term potentiation (Fig. 1E). A significant decrease in firing rate and a significant increase in the burst and network burst frequency were observed upon treatment, similar to previous reports^46^ (Fig. 1F). In addition, we aimed to inhibit the neural activity of the Ngn2 neural co-cultures. To achieve this, we used the S-enantiomer of fluoxetine (SFX), as it has neuro-modulatory effects as well as neurotoxic effects in primary rat cortical neurons.^47^ Adding 10 μM of SFX to the neural co-cultures potently inhibited the neural network activity (Fig. 1E and F). Together, this shows that the neural co-culture model is suitable to detect changes in the network activity.

### NPEVs replicate in neural co-cultures and induce the secretion of interleukin-6 and interleukin-8

**Fig. 2:**
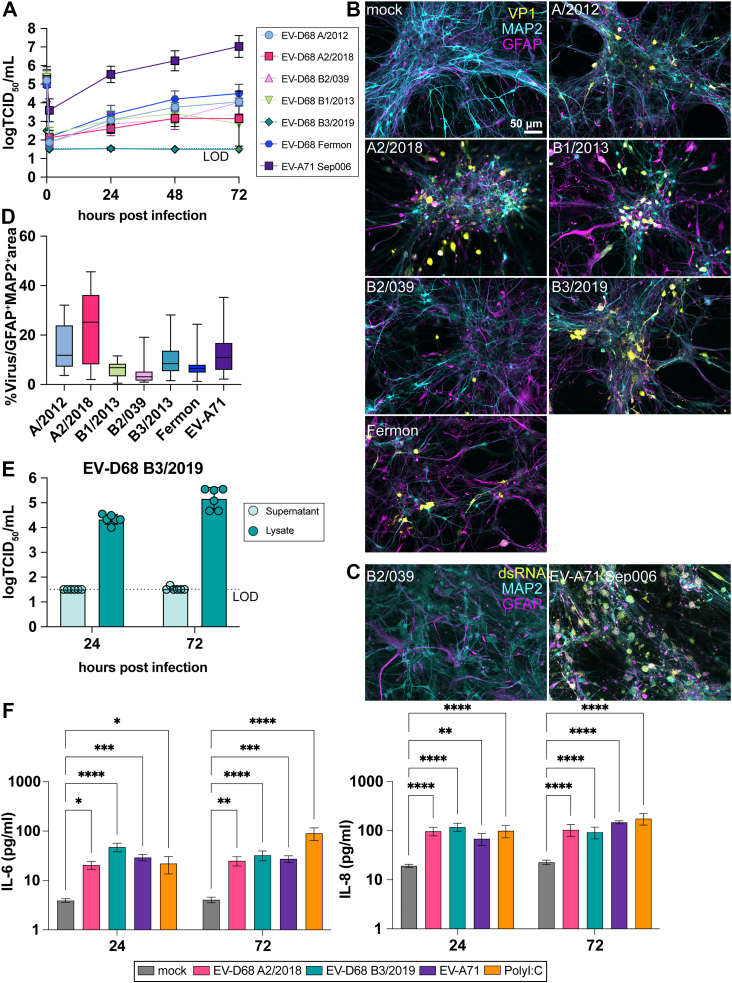
**Enterovirus-D68 and Enterovirus-A71 productively replicate in co-cultures and show similarity in their cell tropism.** (A) Neural co-cultures were infected with different EV-D68 strains and the EV-A71 strain Sep006 from the C4 genotype at an MOI of 0.1. Viral titres were determined in the supernatant at the indicated timepoints by endpoint dilution, with the first data point being the back-titration of the inoculum. Replication kinetics were performed in technical triplicates in three independent experiments and data represented show the mean with 95% CIs. (B) At 24 hpi, the co-cultures were fixed and stained for the presence of EV-D68 structural antigen VP1 (yellow). MAP2 (cyan) was used as a marker for neurons, and astrocytes were identified by staining for GFAP (magenta). (C) At 24 hpi, EV-A71 inoculated co-cultures were fixed and stained for the presence of dsRNA, a marker for active EV-A71 replication (yellow), astrocytes (GFAP, magenta) and neurons (MAP2, cyan). The immunofluorescence data shown are representative examples from three independent experiments for each culture condition. Maximum intensity projections of Z-stacks are displayed. (D) Quantification of infection percentage over MAP2 and GFAP positive area. Bars represent lower (Q1) and upper (Q3) quartile with the median values of the percentage of the virus infection as a line in the box, and whiskers indicate minimum and maximum values within 1·5 times IQR from Q1 and Q3. Data are derived from three independent experiments from which ten images per experiment were taken. (E) Neural Co-cultures were infected with EV-D68 B3/2019 at MOI of 0.1. Viral titres were determined in the supernatant and lysates of infected co-cultures. Data shown represent the mean values ± 95% CIs from three independent experiments, each performed in technical duplicate. (F) At 24 and 72 hpi, cytokine protein concentrations were measured in the supernatant of infected neural co-cultures using a LEGENDplex cytokines assay. Data represented here show data points of cytokines derived from three independent experiments performed in biological triplicates, and the mean with 95% CIs is depicted. Statistical significance was determined with a One-Way ANOVA, comparing the secretion of cytokines induced by viruses or polyI:C stimulation to the mock condition for each timepoint. Asterisks indicate statistical significance (∗*P* < 0.05, ∗∗*P* < 0.01, ∗∗∗*P* < 0.001, ∗∗∗∗*P* < 0.0001). Abbreviations: EV, enterovirus; MOI, multiplicity of infection; SEM, standard error of the main; hpi, hours post inoculation; VP1, viral protein 1; MAP2, microtubule-associated protein 2; GFAP, glial fibrillary acidic protein; dsRNA, double-stranded RNA.

To study the neurotropism and replication kinetics, neural co-cultures were inoculated with EV-D68 strains A/2012, A2/2018, B1/2013, B3/2019, the cell-culture adapted B2/039,^51^ and a recombinant Fermon virus, as well as a recombinant EV-A71 Sep006 from genotype C4 at a MOI of 0.1. At 1-, 24-, 48-, and 72-h post inoculation (hpi), viral titres were determined in the supernatants by endpoint dilution. All viruses, except EV-D68 B3/2019, released infectious virus particles into the supernatant, suggesting efficient replication in the neural co-cultures (Fig. 2A). We observed that, despite comparable EV-D68 and EV-A71 inputs, EV-A71 showed a 1.5 log higher virus titre at 1 hpi after washing the cultures three times. Overall, EV-A71 Sep006 replicated more efficiently compared to all EV-D68 strains based on an area under the curve analysis after adjusting for the higher virus titre 1 hpi (Supplementary Fig. S2A). To determine the cellular tropism of EV-D68, neural co-cultures were fixed at 24 and 72 hpi and stained for the structural protein VP1 (Fig. 2B and Supplementary Fig. S3A). The cell tropism of EV-A71 was visualised using double-stranded RNA, as a marker for active viral replication (Fig. 2C and Supplementary Fig. S3B). All EV-D68 and EV-A71 strains infected MAP2^+^ or GFAP^+^ cells (Fig. 2D, Supplementary Fig. S4A, B). Even though we did not detect EV-D68 B3/2019 titres in the supernatants, virus-infected MAP2^+^ cells were detected. To confirm the intracellular replication of EV-D68 B3/2019 in Ngn2 neural co-cultures, we subjected infected cells to freeze–thaw cycles and detected higher viral titres in the lysates at 72 h compared to 24 h (Fig. 2E). This suggests that EV-D68 B3/2019 is able to produce infectious progeny virus intracellularly. Lastly, we measured cytokine release in infected neural co-cultures. As a positive control, we exposed neural co-cultures to the Toll-like receptor 3 agonist polyinosinic:polycytidylic acid (polyI:C), mimicking double-stranded RNA. This showed that EV-D68 A2/2019, EV-D68 B3/2019 and EV-A71 were able to induce the secretion of interleukin-6 and interleukin-8 while polyI:C stimulation showed a broader profile of cytokine secretion (Fig. 2F, Supplementary Fig. S5A and B). Taken together, all NPEVs showed efficient replication in Ngn2 neural co-cultures, infecting both MAP2^+^ neurons and GFAP^+^ astrocytes, although B3/2019 was impaired in viral release. NPEV infection resulted in the secretion of interleukin-6 and interleukin-8 in neural co-cultures.

**Fig. 3:**
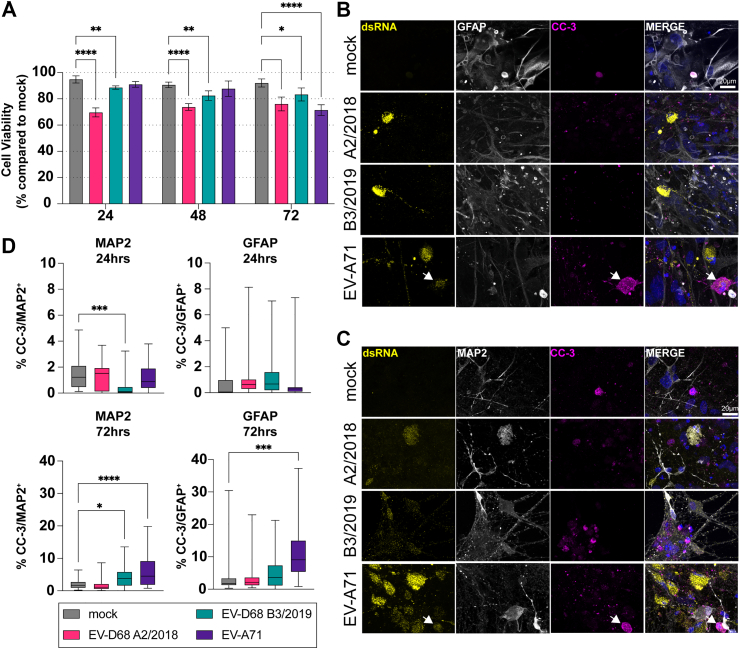
**Enterovirus infection induces cell death in neural co-cultures.** Neural co-cultures were infected with EV-D68 or EV-A71 at an MOI of 1. (A) Bar graphs showing percent cell viability after infection with EV-D68 A2/2018, EV-D68 B3/2019 and EV-A71 Sep006 as measured by amount of LDH released into cell culture supernatant at indicated time points. Data are derived from three independent experiments performed in biological triplicates. Statistical significance was determined with a One-Way ANOVA, comparing the LDH release in virus infected samples to untreated mock control at each timepoint. (B) At 72 h post infection (hpi), neural cultures were fixed and stained for double-stranded RNA (dsRNA; yellow) as a marker of viral infection, for the astrocytic marker GFAP (grey) and for cleaved caspase-3 (CC-3; magenta). Cell nuclei were counterstained with Hoechst (blue). (C) At 72 hpi neural cultures were fixed and stained for dsRNA (yellow), for the neuronal marker MAP2 (grey) and for cleaved caspase-3 (CC-3; magenta) as a marker of apoptosis. Immunofluorescent data shown are representative examples from three independent experiments (B,C). (D) Quantification of CC-3 percentage over MAP2 and GFAP positive areas. Bars represent lower (Q1) and upper (Q3) quartile with the median values of the percentage of CC-3 as a line in the box, and whiskers indicate minimum and maximum values within 1·5 times IQR from Q1 and Q3. Data are derived from three independent experiments from which ten images per experiment were taken. Statistical significance was determined with a One-Way ANOVA, comparing the CC-3 induction in virus infected samples to untreated mock control. Asterisks indicate statistical significance (∗*P* < 0.05, ∗∗*P* < 0.01, ∗∗∗*P* < 0.001, ∗∗∗∗*P* < 0.0001). Abbreviations: EV, enterovirus; MOI, multiplicity of infection; hpi, hours post inoculation; VP1, viral protein 1; dsRNA, double-stranded RNA; MAP2, microtubule-associated protein 2; GFAP, glial fibrillary acidic protein; CC-3, cleaved caspase-3.

Next, we investigated whether NPEV infection resulted in cellular damage. Cell damage was assessed by measuring lactate dehydrogenase (LDH) release into the cell culture supernatant. LDH is a cytoplasmic enzyme that is released extracellularly upon loss of cellular membrane integrity. This showed that infection with EV-D68 A2/2018 and EV-D68 B3/2019 reduced the cell viability significantly already 24 h post infection, while EV-A71 only at 72 hpi (Fig. 3A). Next, we assessed whether NPEV infection induced apoptosis in GFAP^+^ astrocytes or MAP2^+^ neurons. Therefore, we inoculated with a higher MOI and stained for the apoptosis marker cleaved caspase-3 (CC-3) at 24 or 72 hpi, respectively. We detected co-localisation of CC-3^+^ and MAP2^+^ cells occasionally in mock or virus-inoculated cultures. Neither neurons nor astrocytes expressing dsRNA as a marker for virus replication showed co-localisation with CC-3 upon EV-D68 infection similar to what we have observed with other viruses previously (Fig. 3B and C).^43^ In EV-A71 infected cultures we did occasionally observed CC-3 signal in dsRNA-positive cells. We detected the induction of CC-3 in EV-A71 infected cultures at 72 hpi in MAP2^+^ neurons and GFAP^+^ astrocytes, while with EV-D68 infection, the induction of CC-3 was only observed in MAP2^+^ neurons at 72 hpi with EV-D68 B3/2019 (Fig. 3D).

### NPEV infections exert pleiotropic negative effects on neural electrophysiology

**Fig. 4:**
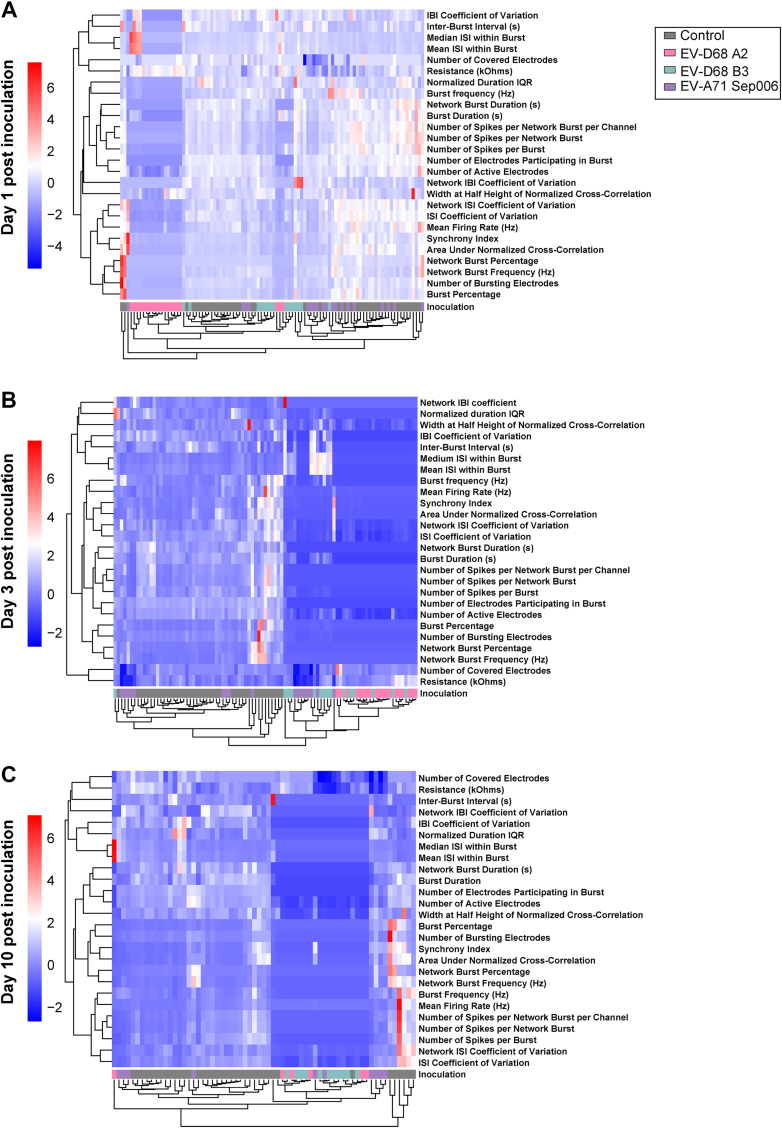
**Heatmap of normalised neural activity data recorded from neural co-cultures inoculated with non-polio enteroviruses.** Neural activity was recorded from neural co-cultures, consisting of Ngn2 neurons and astrocytes, on a MEA platform. Neural co-cultures were mock-inoculated or inoculated with EV-D68 A2/2018, B3/2019, or EV-A71 Sep006 with an MOI of 1. Only data that met inclusion criteria for MEA recordings were used (see Material and Methods). Any experiment that contained *Not Available* values throughout any of the chosen MEA output variables (Table 3) was excluded for PCA analysis, to ensure biological relevance and integrity of the dataset. Data was normalised against baseline recording obtained before inoculation. Data is shown from one (A), three (B), or 10 dpi (C). Rows represent output variables of MEA data and columns represent experimental data from a single well. All MEA data were z-score normalised for each variable across inoculation groups to enable cross-condition comparisons. Colour intensity reflects a positive or negative effect on neural activity, with white to red indicating a more positive effect. Data presented in the heatmap are derived from at least four independent experiments (*n* = 48 for control and *n* = 24 per inoculation group). Abbreviations: Ngn2, Neurogenin-2; MEA, micro-electrode array; EV, enterovirus; MOI, multiplicity of infection; dpi, days post inoculation.

**Fig. 5:**
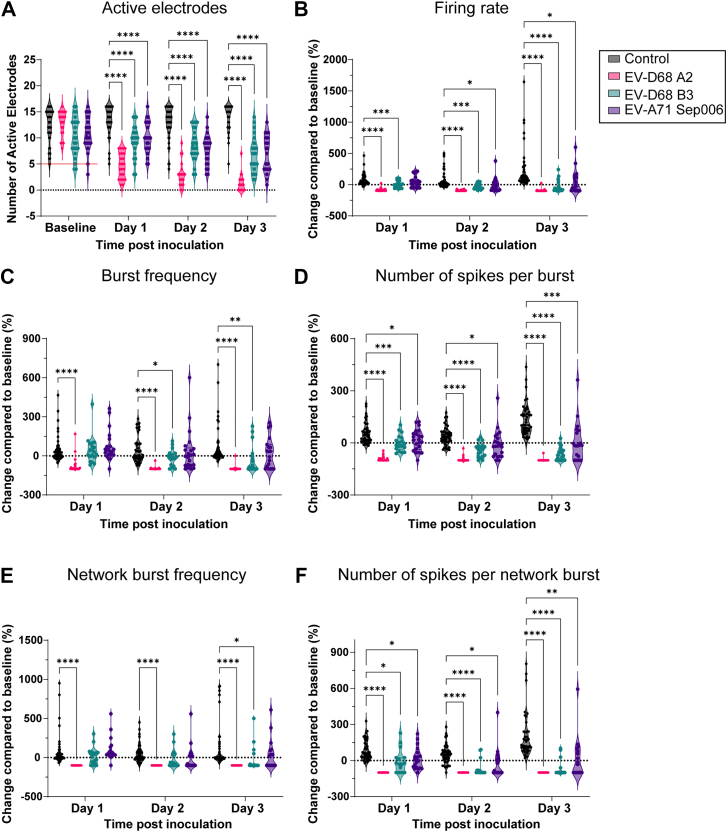
**Enterovirus infection impacts the spontaneous activity of neural co-culture.** Neural co-cultures were inoculated with EV-D68 A2/2018 (pink), B3/2019 (cyan), or EV-A71 Sep006 (purple) with an MOI of 1, and neural activity was measured between one to three dpi. The following parameters were displayed (A) number of active electrodes, where wells were excluded if they contained 5 or less active electrodes at baseline (indicated with a red line); (B) firing rate; (C) burst frequency, (D) number of spikes per burst, (E) network burst frequency and (F) number of spikes per network burst. Data displayed are derived from at least four independent experiments (*n* = 48 for control and *n* = 24 per inoculation group, see exclusion criteria in the Material and Methods section). Statistical significance was calculated with a two-way ANOVA with a Šídák's multiple comparisons post hoc test. Asterisks indicate statistical significance (∗*P* < 0.05, ∗∗*P* < 0.01, ∗∗∗*P* < 0.001, ∗∗∗∗*P* < 0.0001). Abbreviations: EV, enterovirus; MOI, multiplicity of infection; dpi, days post inoculation.

To investigate the neurovirulent potential of currently circulating EV-D68 A2/2018 and B3/2019, as well as EV-A71 Sep006, we inoculated neural co-cultures with an MOI of 1 and measured neural activity using a MEA system. We measured spontaneous and network activity of neural co-cultures over ten days of infection with EV-D68 A2/2018 and B3/2019, and EV-A71 Sep006. A baseline recording of each individual well was performed before inoculation to compare the direct viral effect on the network activity. We performed principal component analysis (PCA) to investigate patterns in our data obtained from the MEA recordings, enabling clear visualisation and in-depth interpretation of complex, high-dimensional data (Fig. 4; Supplementary Fig. S6). To correct for potential virus-induced cell death, cell viability measurements were conducted before each recording. Additionally, we checked whether neural co-cultures had sufficient active electrodes and displayed similar spontaneous activity (Fig. 5A; Supplementary Fig. S7A). At baseline, there were no significant differences observed between designated mock and inoculated groups in the number of covered or active electrodes (Fig. 5A; Supplementary Fig. S7B). All groups displayed similar spontaneous activity represented by firing rate, burst frequency and network burst frequency at baseline before virus inoculation (Supplementary Fig. S7A).

First, we investigated the hierarchical clustering of the output variables of the MEA and the key influencing parameters upon infection (Fig. 4). EV-D68 A2/2018 inoculated cultures clustered together and affected the output variables of the MEA with a mostly negative effect already 1 day post inoculation (dpi; Fig. 4A). The most negative effect was observed in variables such as “Burst Frequency,” “Burst Duration,” “Number of Active Electrodes,” “Number of Spikes per (Network) Burst,” indicating that neural activity is inhibited by EV-D68 A2/2018. PCA analysis confirmed that EV-D68 A2/2018 is more distinct from EV-D68 B3/2019, EV-A71 Sep006, or mock-inoculated controls, suggesting the strongest effect of EV-D68 A2/2018 in impairment of the neural network (Supplementary Fig. S6A). Over the course of infection, the two strains of EV-D68 occupied the same area in the PCA blots indicating a similar negative effect on the neural activity that is distant from EV-A71 at 3 dpi (Fig. 4B and Supplementary Fig. S6B) and 10 dpi (Fig. 4C and Supplementary Fig. S6C). In the mock cultures, a positive effect is observed on most of the MEA output variables, indicating that these cultures are in development and neural activity is increasing (Figs. 4C and 1D). Overall, PCA and heatmap analysis is suggestive that EV-D68 and EV-A71 infections result in adverse changes in the MEA outcomes to varying degrees.

### NPEV infection reduces the network activity of hPSC-derived neural co-cultures

Guided by the PCA analysis, we investigated several parameters in more detail. All three virus strains caused significant reductions in neural activity, but with variations in their temporal patterns. The number of covered electrodes decreased significantly for EV-A71 Sep006 starting at 2 days post-infection and continuing through day 10, while EV-D68 A2/2018 showed this effect from days 5 to 10, and EV-D68 B3/2019 only affected electrode coverage on days 9–10 (Supplementary Fig. S7), possibly reflecting cell death upon infection. All three viruses caused significant decrease in active electrode number between days 1–3 post-infection (Fig. 5A), and these reductions persisted throughout the experimental period of 10 days (Supplementary Figs. S7B, S8A). This suggests that neural activity is inhibited upon virus inoculation by a virus-induced mechanism. This is also shown when the covered electrodes are compared to the active electrodes (Supplementary Fig. S7B). To exclude that impaired neural activity results in cell death, we blocked the electrophysiological activity with TTX and observed the cultures for ten days. While all signal was abolished as soon as 30 min post exposure to TTX, the number of covered electrodes did not change. Furthermore, no obvious cell death was observed in these cultures (LDH assay). This support that reduction of covered electrodes is due to a virus induced change (Supplementary Fig. S9). EV-D68 A2/2018 inoculation has the strongest effect on the number of active electrodes (Supplementary Figs. S7C, S8B), followed by EV-D68 B3/2019 (Supplementary Fig. S7D). Both of the viruses cause cell death measured by the number of covered electrodes, yet with temporal differences. Meanwhile, EV-A71 Sep006 seems to follow a different pattern, where covered and active electrodes are more correlated to each other (Supplementary Fig. S7E). We further investigated different variables indicative for spontaneous neural activity. The neural firing rate was significantly reduced in cultures inoculated with EV-D68 A2/2018 and B3/2019 from day 1 to 3 and continued through day 10. In contrast, EV-A71 Sep006 inoculated cultures showed more variation, and decreased firing rates were observed between 2-3 dpi and 8–10 dpi (Fig. 5B, Supplementary Fig. S8C). EV-D68 A2/2018 inoculated cultures showed significant decreases in burst frequency starting from day 1 onwards, while EV-D68 B3/2019 inoculated cultures demonstrated this effect beginning from day 2 onwards; EV-A71 Sep006 did not alter the burst frequency (Fig. 5C; Supplementary Fig. S8D). All viruses significantly decreased the number of spikes per burst beginning at 1 dpi and persisting through 10 dpi (Fig. 5D; Supplementary Fig. S8E). Overall, these results indicate that while all three virus strains impair neural function, EV-D68 A2/2018 and B3/2019 strains produce more sustained and consistent neurotoxic effects across multiple parameters compared to EV-A71 Sep006.

To further characterise viral effects on neural network activity, we analysed network burst parameters, including network burst frequency and spike density within bursts. Network burst frequency was significantly reduced in EV-D68 A2/2018 inoculated cultures beginning at 1 dpi, while in EV-D68 B3/2019 inoculated cultures this effect started 3 dpi and, in both cases, persisted until 10 dpi. EV-A71 Sep006 did not alter the network burst frequency (Fig. 5E; Supplementary Fig. S8F). Analysis of spikes per network burst revealed that all three virus strains significantly decreased the number of spikes per burst during the early infection period (1–3 dpi) (Fig. 5F). However, the temporal patterns diverged during later time points. EV-D68 A2/2018 and B3/2019 inoculated cultures significantly reduced spike counts per burst throughout the entire 10-day observation period (Supplementary Fig. S8G). In contrast, EV-A71 Sep006 inoculated cultures showed significant reductions occurring only at 5 dpi and from 7 to 10 dpi. Taken together, these findings indicate that EV-D68 and EV-A71 inoculation disrupt coordinated network activity, and they exhibit distinct temporal profiles of network dysfunction. The EV-D68 strains A2/2018 and B3/2019 showed a more pronounced effect on inhibiting the neural network parameters compared to EV-A71. Even though the onset of inhibition differed between EV-D68 and EV-A71, the same parameters were affected over time.

## Discussion

The re-emerging NPEV EV-D68 and EV-A71 are major causes of viral encephalitis, yet their underlying neuropathogenesis remains poorly understood.^1^^,^^52^^,^^53^ In this study, we investigated the replication efficiency and cellular tropism of various EV-D68 and EV-A71 strains, as well as their impact on spontaneous activity, using a physiologically relevant human neural co-culture model consisting of hPSC-derived excitatory cortical Ngn2 neurons and astrocytes. Reduction of spontaneous neural activity and decreased network activity were more pronounced in EV-D68 inoculated cultures compared to EV-A71.

EV-D68 and EV-A71 showed similarities in their cellular neurotropism. We detected that EV-D68 and EV-A71 infected both MAP2^+^ neurons and GFAP^+^ astrocytes. EV-A71 showed a more pronounced astrocyte tropism compared to most of the EV-D68 strains similar to a recent report in a human assembloid model.^54^ It has been shown that EV-D68 infects human astrocytes and mouse astrocytes,^54^, ^55^, ^56^ but not astrocytes in a primary rat neuronal model.^37^ EV-A71 has been detected in neurons, astrocytes, as well as neuron progenitor cells.^26^, ^27^, ^28^^,^^54^^,^^57^ EV-A71 and all EV-D68 viruses, except EV-D68 B3/2019, were able to replicate in neural co-cultures productively. This is in line with previous studies performed in primary rat cortical neurons, a neuron-like cell line and hPSC-derived forebrain, and cerebral organoids.^37^^,^^56^^,^^58^^,^^59^ Similar to other studies, our data also confirm that the ability of different EV-D68 clades to replicate efficiently in neurons is not a recently acquired phenotype, as older isolates also replicate productively.^56^^,^^60^^,^^61^ Our results suggest that all EV-D68 strains are able to release viral progeny into the supernatants, except EV-D68 B3/2019 which was impaired in the release of infectious virus particles. However, in both cases, EV-D68 viruses were able to spread through the cultures over the course of the experiments. This potentially indicates differences in the spreading or release mechanism between EV-D68 strains (e.g. transsynaptic spread or extracellular vesicles release).^62^ Whether this affects the neurovirulent potential of these viruses is currently not understood.

The reduction in the number of covered electrodes and LDH assays suggest that all three viruses induce cell death. EV-D68 A2/2018 showed the strongest reduction in cell viability compared to EV-D68 B3/2019 and EV-A71. Our data suggest that EV-A71 induced the cleavage of caspase-3 in infected cultures 72 h post infection. While EV-D68 B3/2019 induced cleaved caspase-3 in MAP2^+^ neurons, we did not observe this for EV-D68 A2/2018 at any timepoint. This likely suggest an alternative cell death pathway for EV-D68. Similar results have been obtained in a human assembloid model of enterovirus infection, where EV-A71 induced CC-3 cleavage much stronger than EV-D68.^54^ Whether EV-A71 is inducing other forms of cell death in our model remains to be established. It has been shown that EV-A71 is able to induce cell death through apoptosis, pyroptosis, and ferroptosis in different neural cultures,^63^, ^64^, ^65^, ^66^, ^67^ while EV-D68 induced a necrosis-like cell death in primary rat neuronal model.^37^ Neuronal cell death might result from the impairment of neurotransmission or be directly induced by infection. Our data point towards virus induction of cell death, since complete abolishment of neurotransmission in our model with TTX did not affect cell viability (Supplementary Fig. S9).

NPEV infection affected both spontaneous neuronal activity and overall neural network function. EV-D68 A2/2018 induced a rapid shut-down of neural activity. EV-D68 B3/2019 followed this trend, although more slowly and less extensively. Despite the significant differences in the replication between EV-A2/2018 and B3/2019, both viruses produced a sustained and consistent neurotoxic effect. This aligns with earlier research, which found that four different EV-D68 viruses disturb the spontaneous neural activity by reducing the number of spikes and bursts at 24–48 hpi in rat primary neuron cultures. However, this study did not show data on changes in the neural network activity.^37^ We here show that synchronous network activity (network burst frequency) is also reduced upon NPEV infection. EV-A71 Sep006 showed a slower temporal kinetics in impairing neural activity, despite more efficient replication in the neural co-cultures. This suggests that the neurotoxic effects are not determined by viral load but are rather virus-specific.

Our hPSC-derived neural co-culture model provides a valuable platform for studying neuronal activity during viral infection and other brain disorders, with the potential for scalable and reproducible applications.^68^^,^^69^ However, several limitations of our model should be discussed. One technical observation of a consistent dip in neural network activity at day 27 remains unexplained. The neuron-astrocyte co-culture does not fully recapitulate the cellular diversity, structural organisation, immune interactions, and network complexity of the human brain. For example, our model does not contain any inhibitory neurons. Yet, we do observe similar outcomes as in a primary rat neuron model where both excitatory and inhibitory neurons were infected with EV-D68.^37^ Additionally, because of the lack of immune competence in our model, we cannot investigate the full range of brain inflammatory response as reported in EV-induced human encephalitis cases.^27^^,^^29^^,^^30^^,^^67^ Induction of interferons plays an important role in response to viral infection, however we did not observe an induction of type-I (IFNα2, IFNβ), type-II (IFNγ) or type-III (IFNλ1, IFNλ2) interferon but the secretion of pro-inflammatory cytokines (interleukin-6 and -8) was detected in our model with all NPEVs tested (Fig. 2F, Supplementary Fig. S5A). Which inflammatory responses contribute to the development of EV-induced encephalitis and whether this differs between enterovirus species is currently unknown. Omics-based studies comparing changes in gene expression or metabolomics can help to shed light on the molecular mechanisms responsible for the decrease in neurotransmission for EV-D68 and EV-A71.^70^ Non-lytic release observed here with EV-D68 B3 strain has been reported for many enterovirus species and additional experiments are needed to define the exact spreading mechanism of B3 in neurons.^62^

Our study demonstrates that NPEV infections have profound effects on the neural network activity providing a link between infection and disturbances in the brain homoeostasis and functionality using a physiologically relevant hPSC-derived neural *in vitro* model. In clinical settings, alterations in brain activity detected by EEG have been reported in patients with enterovirus-induced encephalitis, including diffuse background slowing and focal abnormalities.^71^ However, with limited longitudinal EEG data during symptom development, virus serotype/strain-specific differences cannot be distinguished, and whether focal abnormalities correspond to the site of infection remains unknown. At this stage, we can speculate that these EEG observations may stem from NPEV-induced disruption of neurotransmission, as demonstrated in our model. Additionally, neuronal cell death has been observed in patients diagnosed with NPEV, which aligns with our *in vitro* findings.^72^^,^^73^ Current *in vitro* models cannot represent the full spectrum of neurological complications caused by NPEVs, but the development of additional tools, including 2D and 3D cell models representative of spinal cord and brainstem region, holds significant promise to elucidate neuropathological mechanisms bridging the gap between *in vitro* findings and patient outcomes, ultimately advancing therapeutic development.

## Contributors

FB: data acquisition and analysis (MEA, imaging, virology); methodology, visualisation, data verification, writing original draft.

SSNA: data acquisition and analysis (virology).

AKPDS: data acquisition and analysis (imaging, virology), visualisation.

MP: data analysis (MEA), methodology.

WR: data analysis (MEA), resources, methodology.

AD: data acquisition (virology), methodology.

BM: data acquisition (virology), resources, methodology.

FMSDV: methodology, resources, funding acquisition.

DVR: conceptualisation, supervision, funding acquisition.

KL: conceptualisation, study design, data analysis, data verification, writing original draft, supervision, funding acquisition.

LB: conceptualisation, study design, data acquisition and analysis (MEA, imaging, virology); methodology, resources, visualisation, data verification, writing original draft, supervision, funding acquisition.

All authors contributed to the editing and reviewing of the manuscript. All authors have read and approved the final version of the manuscript.

KL AND LB had access to and validated the underlying data in the manuscript.

## Data sharing statement

The data that support the findings in the study are provided within the article, the figures, tables, or appendix. Raw data or other relevant information are available upon request to the corresponding author.

## Declaration of interests

All authors declare that they have no conflicts of interest.

## Appendix A Supplementary data

Supplement

Primary ab validation

Benavides Cell-Line-Checklist
