# Supplementary material for: Non-polio enterovirus infection and electrophysiological changes in human iPSC-derived neural networks
Source: eBioMedicine. 2026 Mar 12;126:106201. doi: 10.1016/j.ebiom.2026.106201 (PMC12996996; doi:10.1016/j.ebiom.2026.106201)
Supplement: Supplement [file mmc1.docx]

***Supporting Information***

**Non-polio enteroviruses compromise the electrophysiology of a human iPSC-derived neural network**

**Authors:**

Feline F. W. Benavides^1^, Syriam Sooksawasdi Na Ayudhya^1,2,*^, Ashley K. Pereirinha da Silva^1,*^, Mark A. Power^1^, Willemijn F. Rijnink^1^, Auriane Deguergue^3^, Bjoern Meyer^3^, Femke M. S. de Vrij^4^, Debby van Riel^1^, Kristina Lanko^5^, Lisa Bauer^1,#^

Affiliation

^1^ Department of Viroscience, Erasmus MC University Medical Center, Rotterdam, The Netherlands

^2^ Current affiliation: Faculty of Veterinary Science, Prince of Songkla University, Songkhla, Thailand

^3^ Medical Faculty, Institute of Medical Microbiology and Hospital Hygiene, Otto-von-Guericke-University Magdeburg, Magdeburg, Germany

^4^ Department of Psychiatry, Erasmus MC University Medical Center, Rotterdam, The Netherlands

^5^ Department of Clinical Genetics, Erasmus MC University Medical Center, Rotterdam, The Netherlands

^*^ authors contributed equally

# corresponding author: [l.bauer@erasmusmc.nl](mailto:l.buaer@erasmusmc.nl)

**Supplementary Figures**


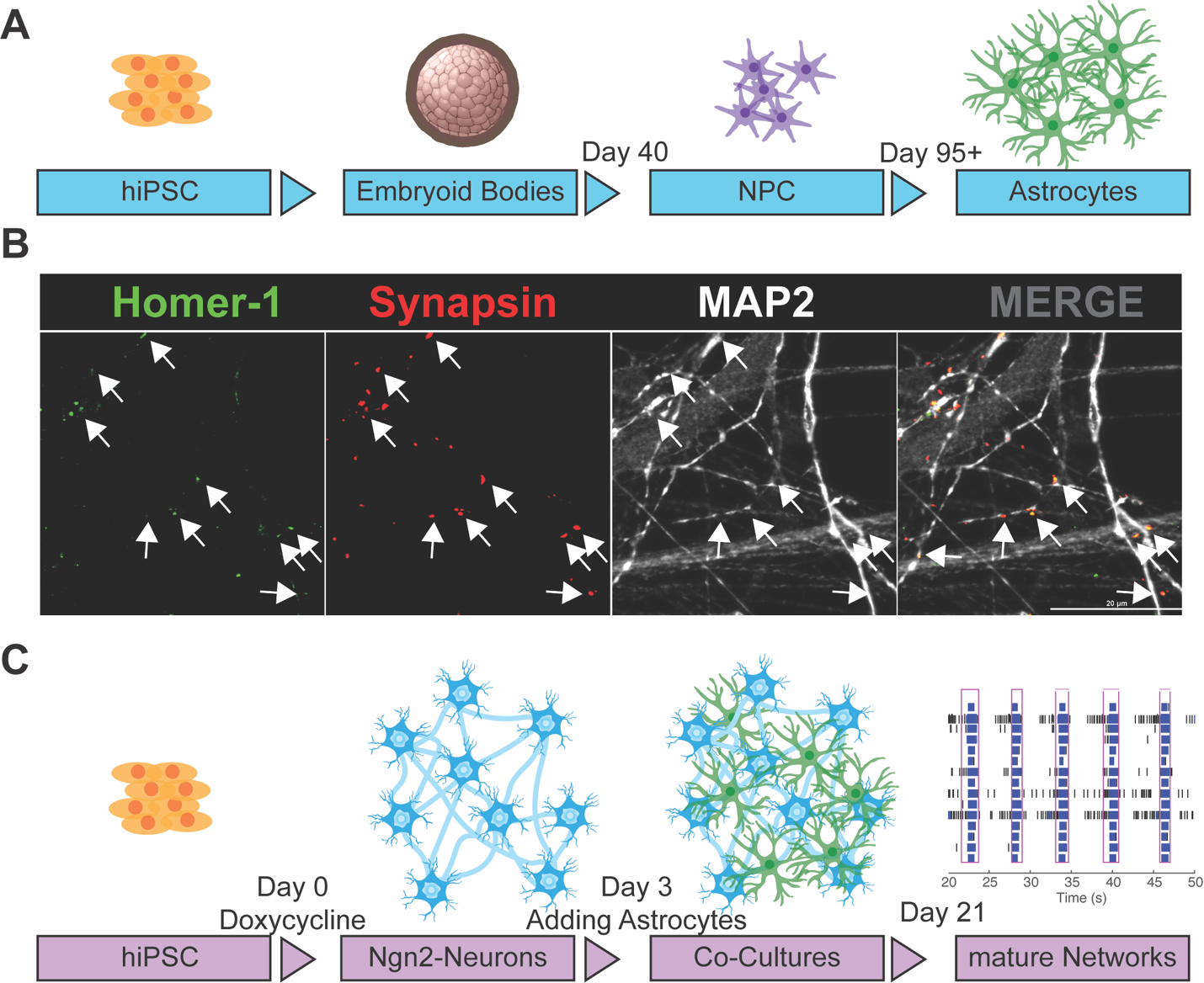


**Supplementary Fig 1. Overview of differentiation strategy.** (A) hiPSC are differentiated into NPCs and further into a pure culture of astrocytes as previously described1. (B) Immunofluorescent staining for an overview of functional synapses was performed against MAP2 (grey), synapsin-1 (red, marker for pre-synaptic density), and Homer 1 (green, marker for post-synaptic density) at DIV 22. (C) hiPSCs are differentiated into excitatory neurons by inducing overexpression of Ngn2 with Doxycycline. At day 3, astrocytes are added to the Ngn2-neurons in a 1:1 ratio and matured until DIV 21. From DIV 21, the Ngn2 neural co-cultures are electrophysiological active and used in experiments. Abbreviations: hiPSC = human induced pluripotent stem cells; NPC = neuron progenitor cells; Ngn2 = Neurogenin2; DIV = days in vitro;


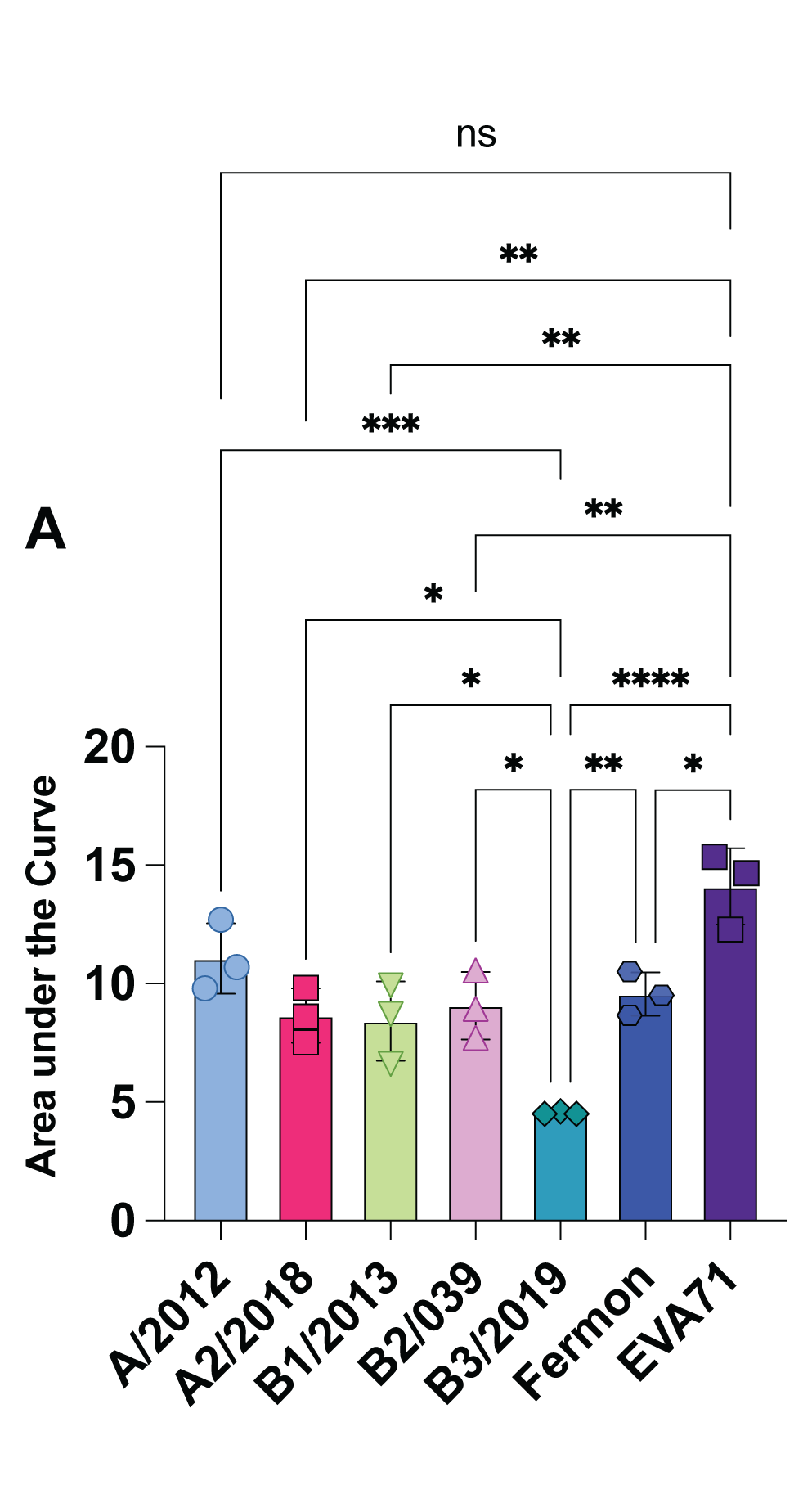


**Supplementary Fig 2. Area under the curve analysis of titres produced by Enterovirus-D68 and Enterovirus-A71 viruses in neural co-cultures.** (A) To evaluate statistical significances between the replication kinetics of EV-D68 and EV-A71 viruses in neural co-cultures, the area under the curve was calculated. The area under the curve for EV-A71 was correct for the higher input of EV-A71. Data displayed represent mean with standard deviation from three independent experiments. Differences in the growth curve between the different viruses were analysed statistically with a One-Way Anova with a Tukey’s post-hoc test to compare different groups.


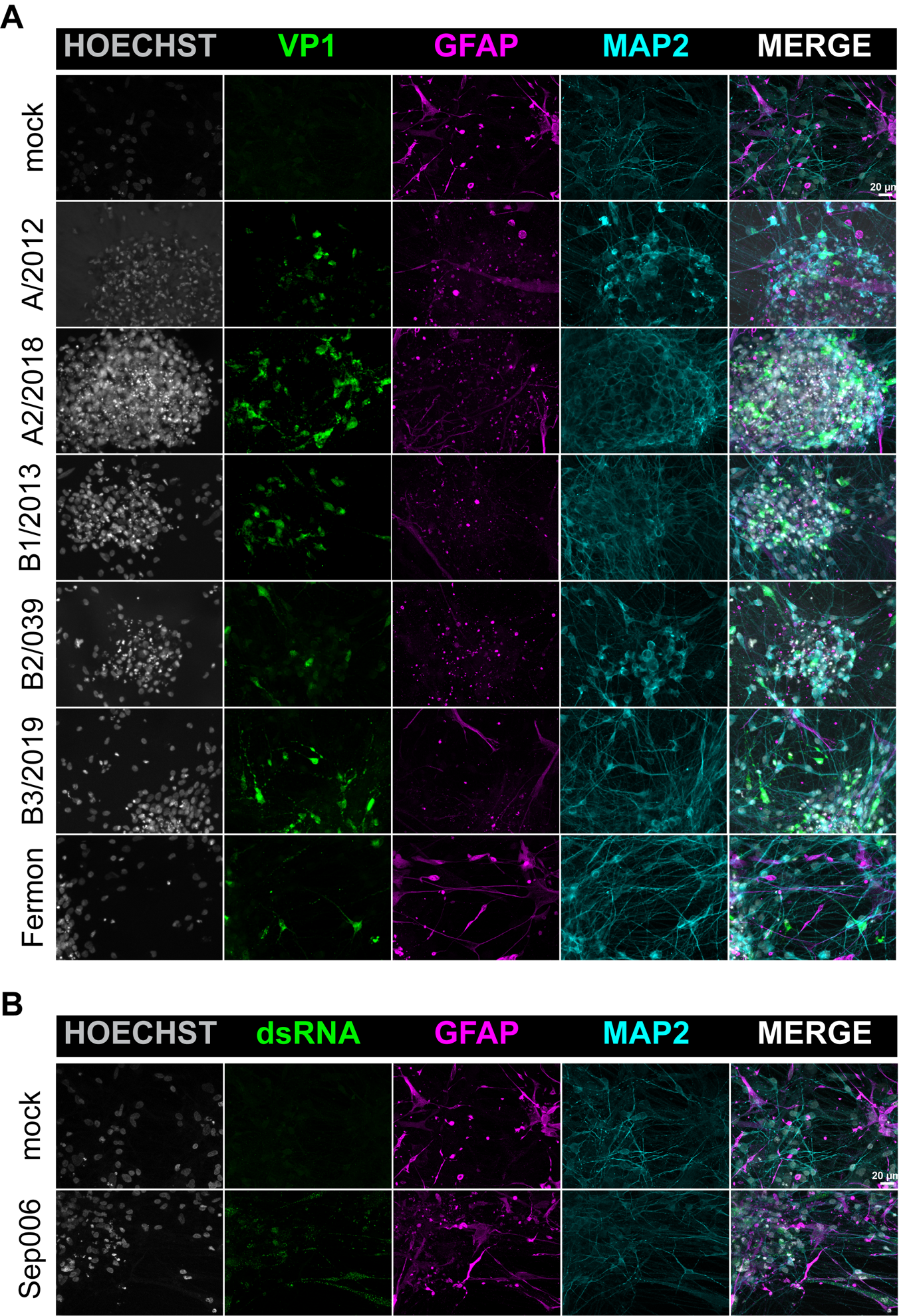


**Supplementary Fig 3.** **Enterovirus-D68 and Enterovirus-A71 infect neural co-cultures and show similarity in their cell tropism.** (A) 72 hpi, the neural co-cultures were fixed and stained for the presence of EV-D68 structural antigen VP1 (green). MAP2 (cyan) was used as a marker for neurons, astrocytes were identified by staining for GFAP (magenta). Cells were counterstained with Hoechst (grey) to visualize the nuclei. (B) 72 hpi, the co-cultures were fixed and stained for the presence of double stranded RNA, a marker for active EV-A71 replication (green), the astrocytic marker GFAP, neuron marker MAP2 and Hoechst. Immunofluorescence data shown are representative examples from three independent experiments. Maximum intensity projections of Z-stacks are displayed. Abbreviations: hpi = hours post inoculation; EV = enterovirus; VP1 = viral protein 1; MAP2 = microtubule-associated protein 2; GFAP = glial fibrillary acidic protein; dsRNA = double-stranded RNA


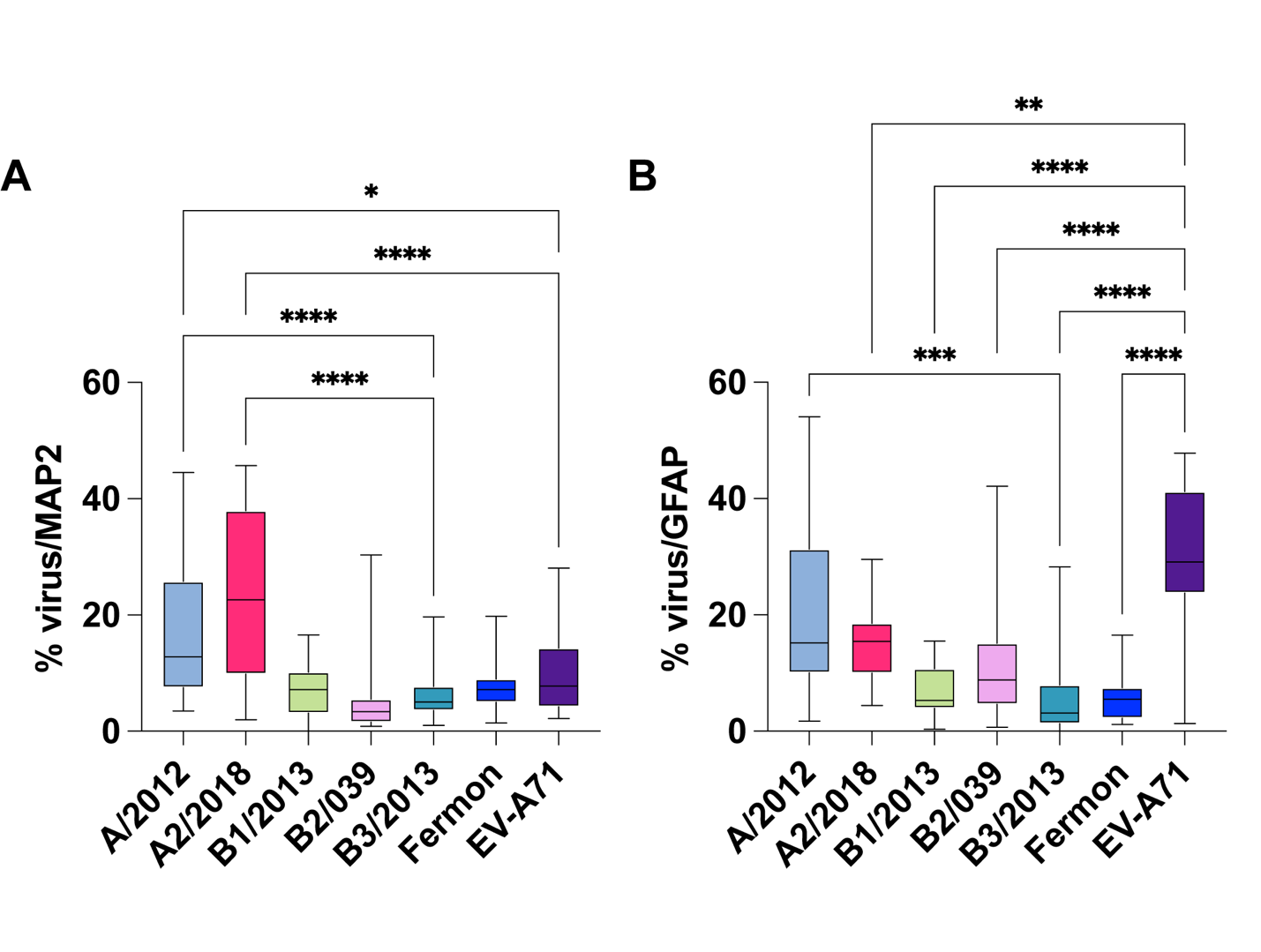


**Supplementary Fig 4. Cell tropism of EV-D68 and EV-A71 in neural co-cultures.** Quantification of infection percentage of (A) MAP2^+^ and (B) GFAP^+^ areas. Bars represent lower (Q1) and upper (Q3) quartile with the median values of the percentage of the virus infection as a line in the box, and whiskers indicate minimum and maximum values within 1·5 times IQR from Q1 and Q3. Data are derived from three independent experiments from which ten images per experiment were taken.


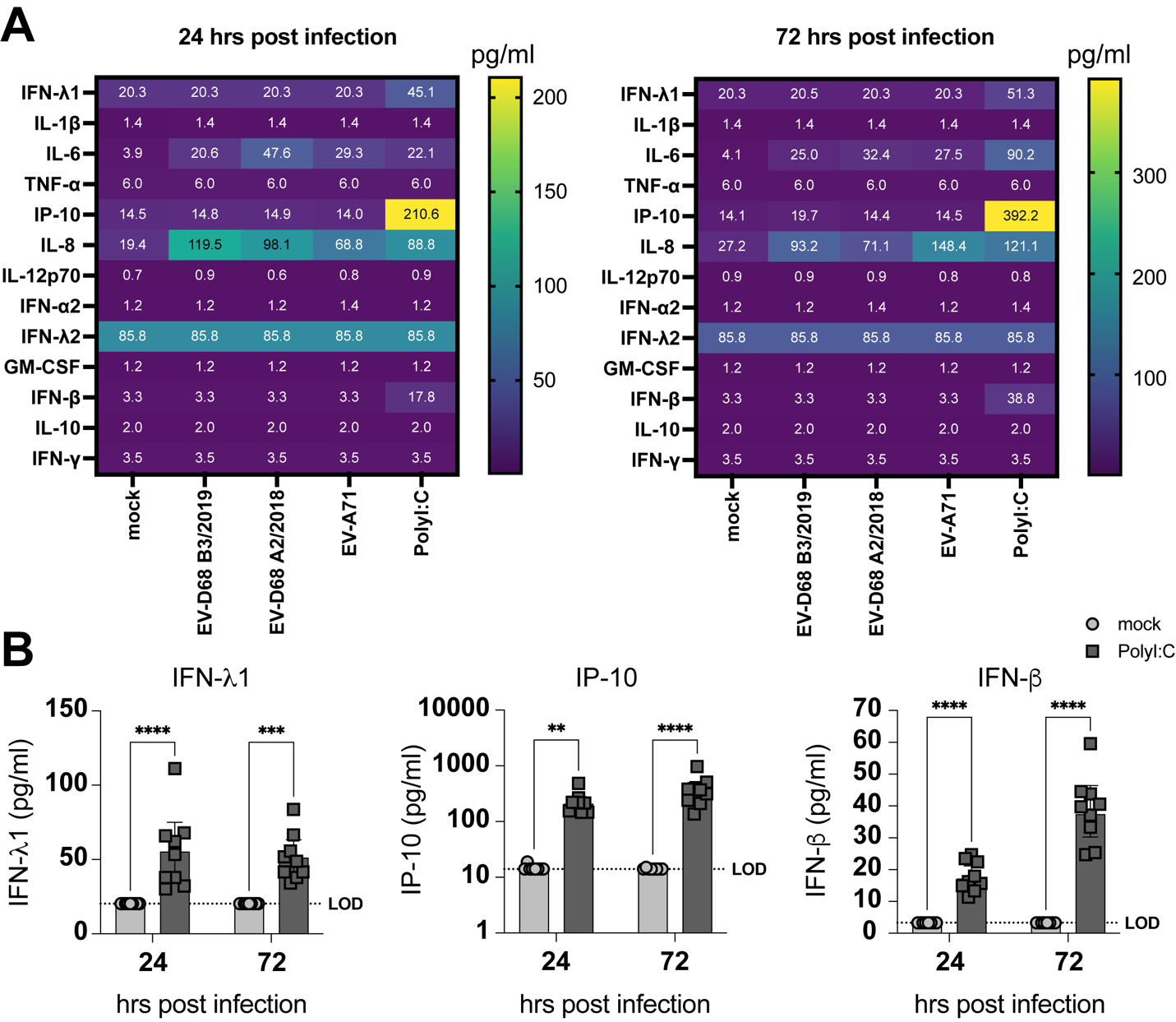


**Supplementary Fig 5. Cytokine profiling in neural co-cultures infected with non-polio enteroviruses and upon stimulation with polyI:C. (**A) Neural co-cultures were inoculated with EV-D68 A2/2019, EV-D68 B3/2019 and EV-A71 Sep006 or stimulated with the TLR-3 agonist polyI:C. At 24 and 72 hrs post-infection/stimulation cytokines were measured in the supernatants and and protein concentrations were determined using the LEGENDplex assay. (B) Data represented in the heatmap show the mean value of cytokines derived from three independent experiments performed in biological triplicates. (B) PolyI:C stimulation induces the secretion of IFN-l1, IP-10 and IFN-b.

**
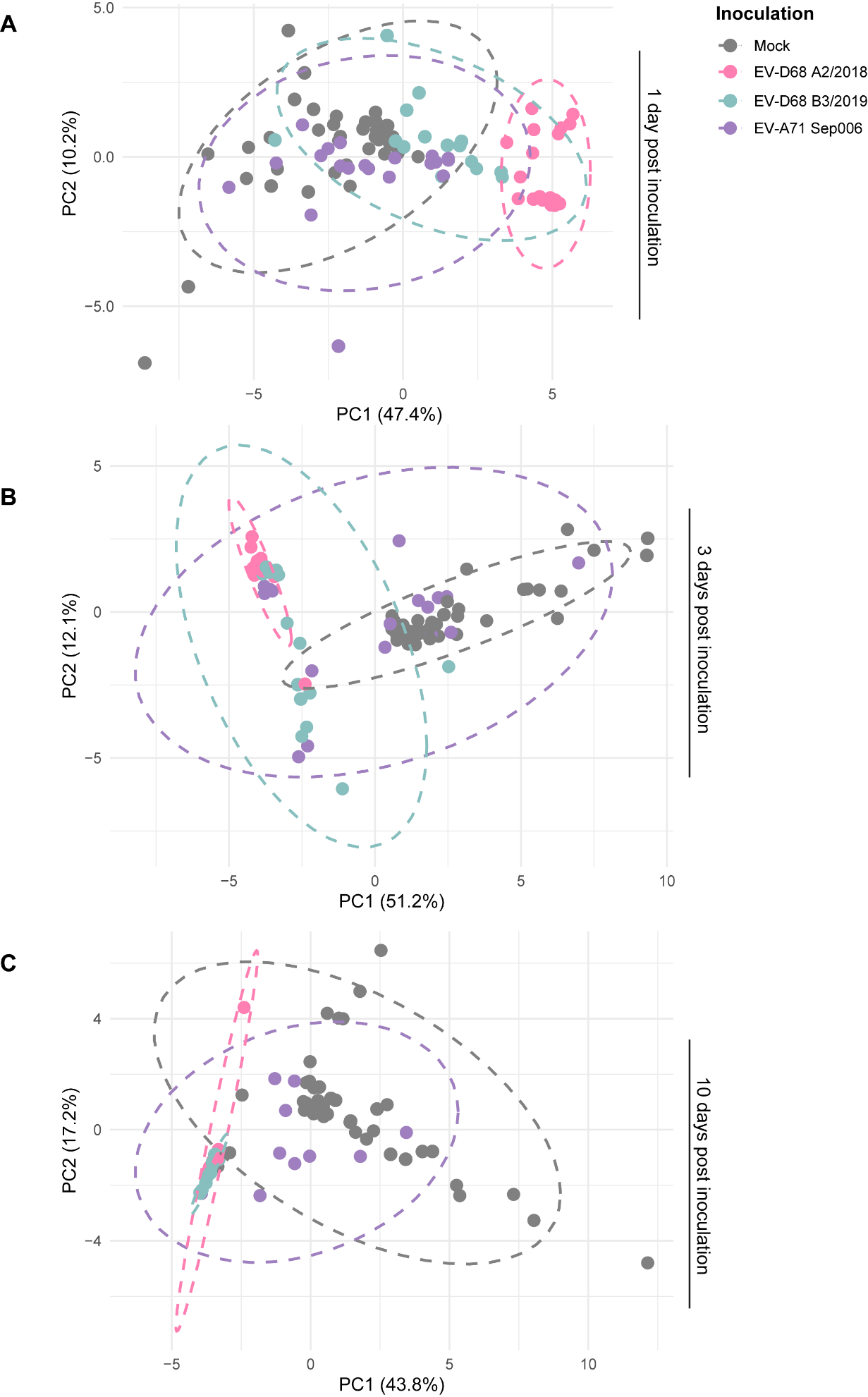
**

**Supplementary Fig 6. Principal Component Analysis (PCA) of neural activity recorded from co-cultures inoculated with Enterovirus-D68 A2, B3 or Enterovirus-A71 Sep006.** PCA was performed on neural data recorded from neural co-cultures, consisting of Ngn2 neurons and astrocytes on a MEA platform. Cultures were mock-inoculated, or with EV-D68 A2/2018, B3/2019 or EV-A71 Sep006 (*n* = 48 for control and *n* = 24 per inoculation group, unless datapoints were excluded based on exclusion criteria as described in the Material and Methods section). PCA plots of one (A), three (B), and ten dpi (C) are shown. The first two principal components (PC1 and PC2), accounting for 47.4% and 10.2% (1 dpi), 51.2% and 12.1% (3 dpi), and 43.8% and 17.2% (10 dpi) of the total variance, respectively, are shown. Each point represents experimental data from a single well, with colours indicating inoculation groups. PCA was conducted on all output variables of MEA data stated in the Material and Methods section. A 95% prediction ellipse is depicted for each group, highlighting differences in neural activity patterns across inoculation groups. Abbreviations: PCA = principal component analysis; Ngn2 = Neurogenin-2; MEA = micro-electrode array; EV = enterovirus; dpi = days post inoculation;


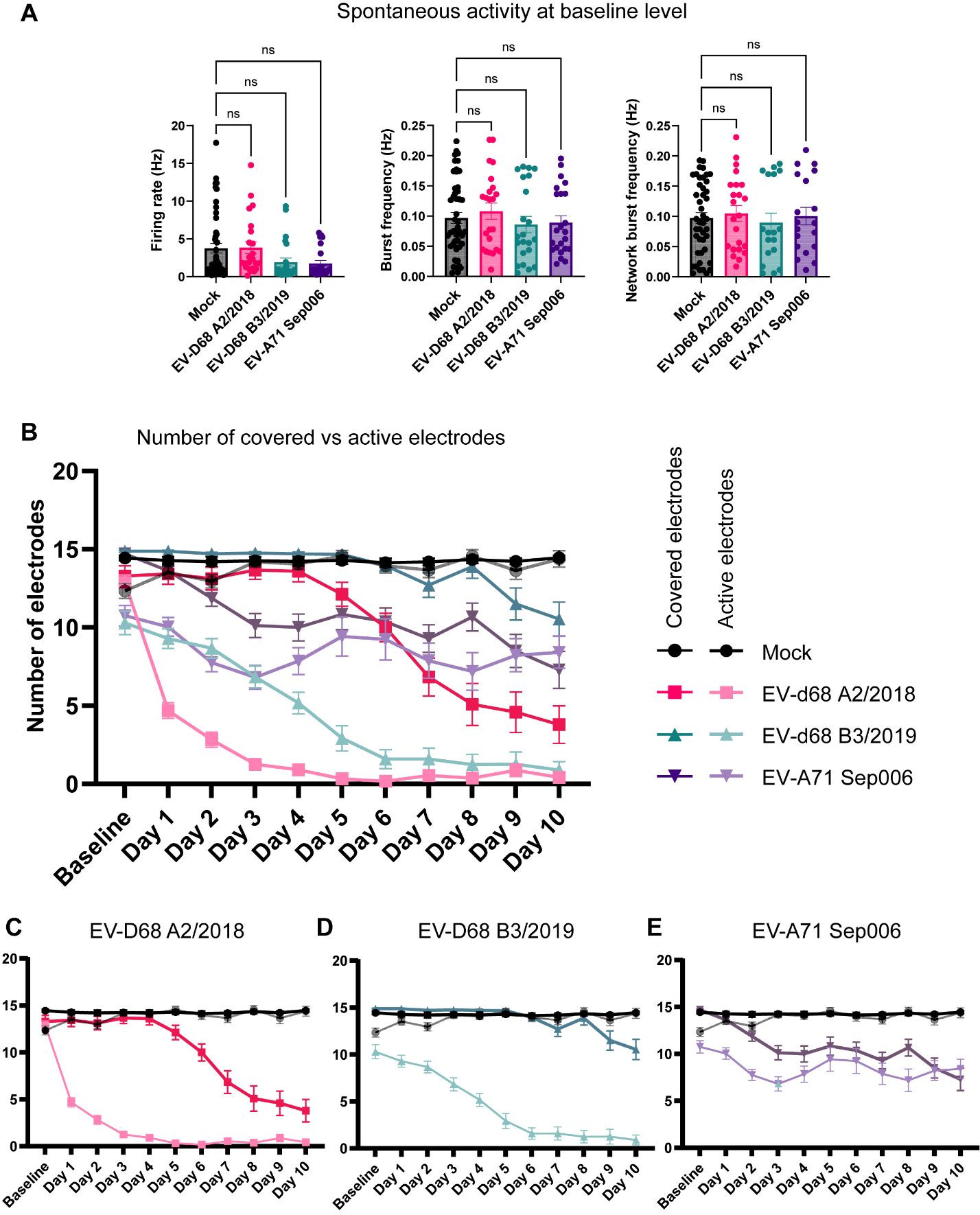


**Supplementary Fig 7. Neural Network activity of cultures. (A)** Firing rate, burst frequency, and network burst frequency at baseline recording. (B) Comparison of covered and active electrodes during the time of infection with the viruses (C) EV-D68 A2/2018, (D) EV-D68 B3/2019 and (E) EV-A71 Sep006.

­­­­
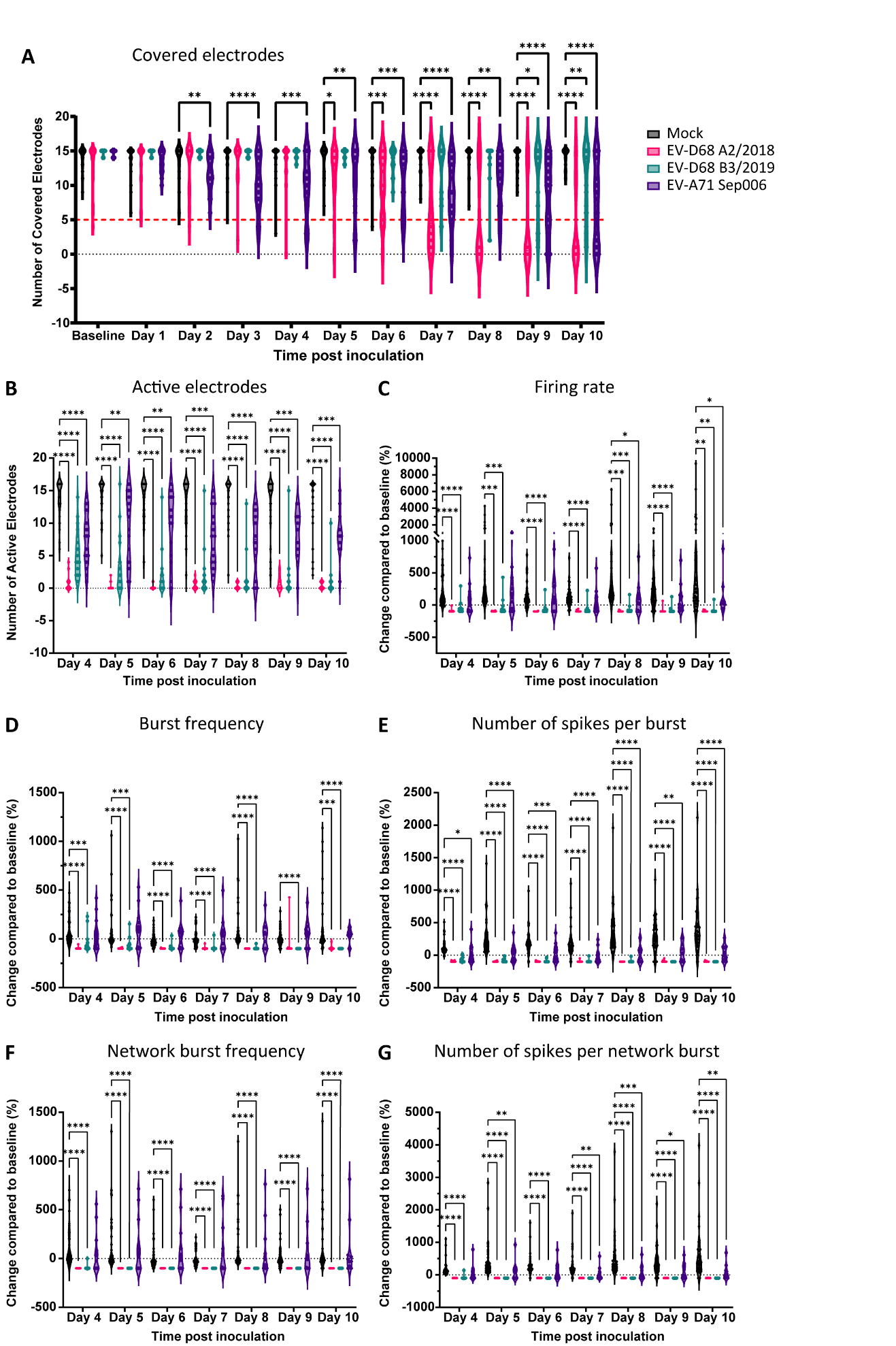


**Supplementary Fig 8. Enterovirus infection impacts the spontaneous activity of co-cultures long-term.** Neural co-cultures were inoculated with EV-D68 A2/2018 (pink), B3/2019 (cyan) or EV-A71 Sep006 (purple) with a MOI of 1, and neural activity was measured between 4 and 10 dpi. The following parameters were displayed (A) number of covered electrodes, where wells were excluded for further analysis if they reached 5 or less covered electrodes throughout the experiment, indicating cell death; (B) number of active electrodes; (C) firing rate; (D) burst frequency, (E) number of spikes per burst, (F) network burst frequency and (G) number of spikes per network burst. Data is displayed from at least four independent experiments performed in six replicates (*n* = 48 for control, and *n* = 24 per inoculation group, unless datapoints were excluded based on exclusion criteria, see Material and Methods). Statistical significance was calculated with a two-way ANOVA with a Šídák's multiple comparisons post hoc test. Asterisks indicate statistical significance (**P*<0.05, ***P*<0.01, ****P*<0.001, *****P*<0.0001). Abbreviations: EV = enterovirus; MOI = multiplicity of infection; dpi = days post inoculation.

**
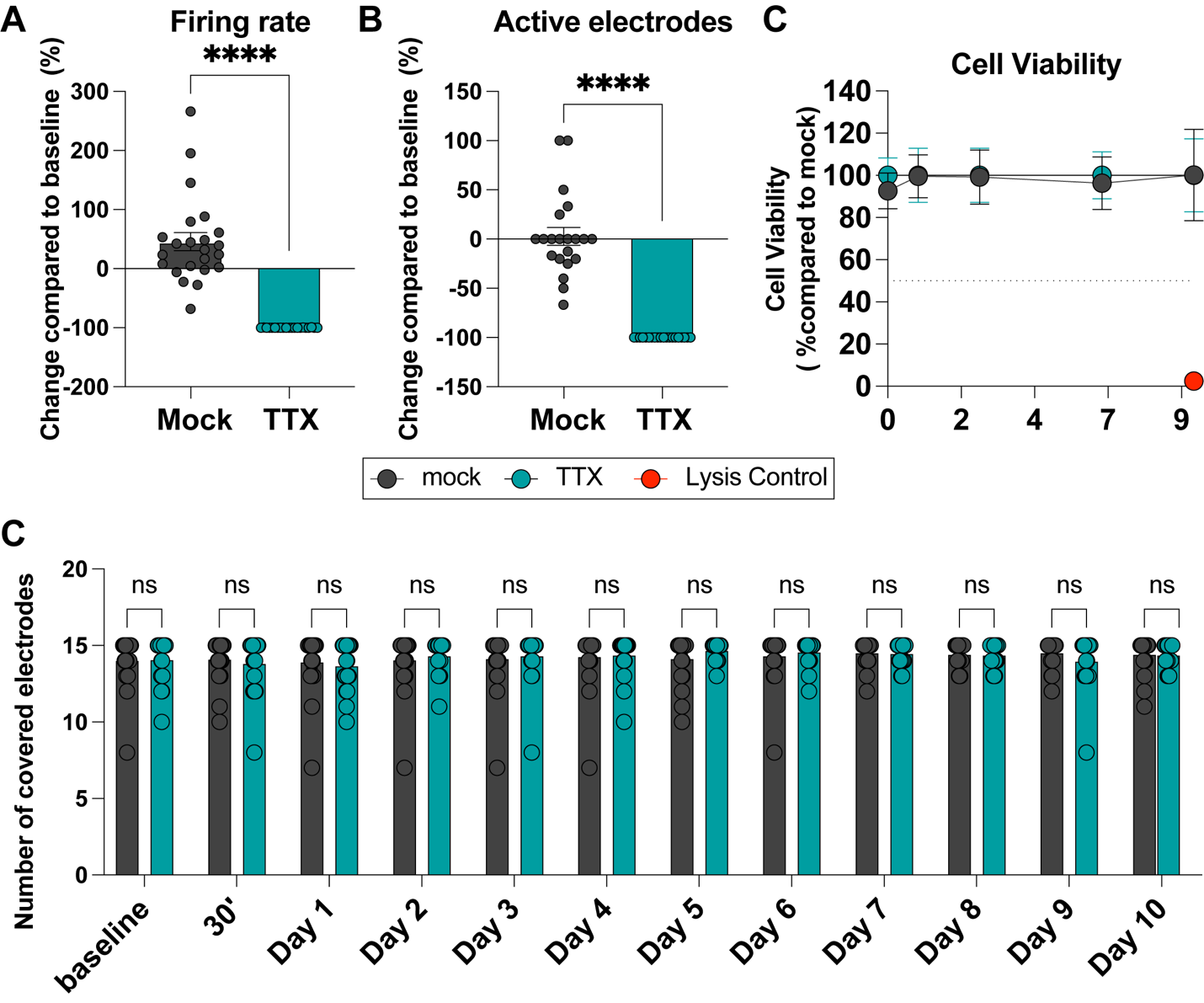
**

**Supplementary Fig 9. Blockage of neurotransmission with tetrodotoxin does not results in cell death.** Neurotransmission of neural co-cultures was blocked by infusion with tetrodotoxin (TTX) with a final concentration of 0.5 µM for 30 minutes. Displayed are (A) the firing rate (A) and (B) number of active electrodes after inhibition with TTX compared to baseline recording before inhibition. (C) The number of covered electrodes is depicted between control or inhibited neural co-cultures over time. Data are depicted as mean ± SEM. Data are derived from four independent experiments (n = 6 per group, unless datapoints were excluded based on exclusion criteria, see Material and Methods).

**References:**

1. Lendemeijer B, Unkel M, Smeenk H, et al. Human Pluripotent Stem Cell-Derived Astrocyte Functionality Compares Favorably with Primary Rat Astrocytes. *eNeuro*. 2024;11(9). doi:10.1523/ENEURO.0148-24.2024
