# Supplementary material for: Non-polio enterovirus infection and electrophysiological changes in human iPSC-derived neural networks
Source: eBioMedicine. 2026 Mar 12;126:106201. doi: 10.1016/j.ebiom.2026.106201 (PMC12996996; doi:10.1016/j.ebiom.2026.106201)
Supplement: Primary ab validation [file mmc2.docx]

| **Antibody** | **Antibody** | Reference | **Manufacturer** | **Catalogue no.** |
| --- | --- | --- | --- | --- |
| Primary | Anti-dsRNA Antibody, clone rJ2,  RRID:AB_2922431 | PMID:40512618 | Jena Bioscience | RNT-SCI-10010200 |
|  | Rabbit anti-EV-D68 VP1,  RRID:AB_2886609 | PMID: 40912913 | GeneTex | 132313 |
|  | Guinea pig anti-MAP2  RRID:AB_2138181 | PMID:40531619 | Synaptic Systems | 188004 |
|  | Mouse anti-GFAP,  RRID:AB_396365 | PMID:40073022 | BD Pharma | 556327 |
|  | Chicken anti-GFAP,  RRID:AB_304558 | PMID:39731735 | Abcam | AB-4674 |
|  | Mouse α-Homer1,  RRID:AB_2619855 | PMID: 39227152 | Synaptic systems | 160011 |
|  | Rabbit α-Synapsin I,  RRID:AB_11042000 | PMID: 39227152 | Synaptic systems | 106103 |
|  | Rabbit Cleaved Caspase-3,  RRID:AB_2341188 | PMID: 34160239 | Cell Signalling | 9661 |
